# Comparison of Cognitive Intervention Strategies for Individuals With Alzheimer’s Disease: A Systematic Review and Network Meta-analysis

**DOI:** 10.1007/s11065-023-09584-5

**Published:** 2023-03-16

**Authors:** Chunchen Xiang, Yumei Zhang

**Affiliations:** 1 Department of Neurology, Beijing Tiantan Hospital, Capital Medical University, Beijing, China; 2 Department of Rehabilitation Medicine, Beijing Tiantan Hospital, Capital Medical University, Beijing, 100070 China; 3 Center of Stroke, Beijing Key Laboratory of Translational Medicine for Cerebrovascular Disease, Beijing Institute for Brain Disorders, Beijing, China; 4 China National Clinical Research Center for Neurological Diseases, Beijing, China

**Keywords:** Alzheimer’s disease, Cognitive rehabilitation, Cognitive stimulation, Cognitive training, Network meta-analysis

## Abstract

Accumulating evidence has shown the effectiveness of cognitive interventions, which can be divided into cognitive training (CT), cognitive stimulation (CS), cognitive rehabilitation (CR), and combined interventions (i.e., cognitive interventions combined with other non-pharmacological interventions such as physical exercise), in individuals with Alzheimer’s disease (AD). However, the effectiveness of cognitive interventions varies greatly among studies and more comprehensive studies are required. We aimed to evaluate whether the current evidence shows that cognitive interventions are effective at improving cognition, neuropsychiatric symptoms, depression, quality of life, and basic activities of daily living among individuals with possible or probable AD. Randomized controlled trials of all types of cognitive intervention were identified for inclusion in pairwise and network meta-analyses. There was a moderate and statistically significant post-intervention improvement in global cognition among individuals with AD for all types of cognitive intervention compared to control interventions (39 studies, g = 0.43, 95% CI: 0.28 to 0.58,* p* < 0.01; Q = 102.27, df = 38, *p* < 0.01; I^2^ = 61.97%, τ^2^ = 0.13). Regarding the specific types of cognitive intervention, combined interventions had the highest surface under the cumulative ranking curve (SUCRA) value (90.7%), followed by CT (67.8%), CS (53.4%), and lastly CR (28.9%). Significant effects of cognitive interventions were also found for working memory, verbal memory, verbal fluency, confrontation naming, attention, neuropsychiatric symptoms, basic activities of daily living, and quality of life.

**Supplementary Information:**

The online version contains supplementary material available at 10.1007/s11065-023-09584-5.

## Introduction

With the aging of the global population, Alzheimer’s disease (AD) has become a leading cause of disability and represents an enormous societal burden (Jia et al., 2018). Currently, cholinesterase inhibitors are the primary pharmacological treatment for cognitive symptoms in AD. However, cholinesterase inhibitors have a poor risk-benefit relationship, indicated by frequent discontinuation and mild symptom improvement (Blanco-Silvente et al., 2017). Non-pharmacological interventions can be beneficial for AD prevention and treatment and, importantly, are less likely to cause adverse events (Livingston et al., 2020). For example, cognitive intervention has been recommended for mild cognitive impairment (MCI) in clinical guidelines (Petersen et al., 2018), however, there is insufficient evidence for use of cognitive intervention in individuals with mild to severe dementia (Arvanitakis et al., 2019).

According to Clare and Wood’s research, cognitive interventions can be divided into three categories, including cognitive training (CT), cognitive stimulation (CS), and cognitive rehabilitation (CR: Clare et al., 2003). CT, which involves a standardized task with a range of difficulty levels, aims to improve specific cognitive domains (Bahar-Fuchs et al., 2019; Trebbastoni et al., 2018). CS, which involves a wide range of group-oriented social events, aims to generally improve cognitive function and behavior (Cafferata et al., 2021; Oliveira et al., 2021). CR, which is an individualized method, aims to achieve optimal levels of physical, psychological, and social functioning (Bottino et al., 2005). Although there are many studies on the effectiveness of various cognitive interventions, very few reviews have focused on summarizing the treatment results. Moreover, the possibility of combining CT with non-pharmacological interventions or non-specific cognitive activities, such as physical exercise (Young et al., 2019) or CS (Barban et al., 2016), has been highlighted as a potential approach for improving cognitive function in AD (Gavelin et al., 2021).

Recently, traditional pairwise meta-analysis has been increasingly used to evaluate the efficacy of cognitive interventions on cognitive performance in cognitively healthy older adults (Lampit et al., 2014), individuals with MCI (Liang et al., 2019) and individuals with AD (Bahar-Fuchs et al., 2019; Cafferata et al., 2021; Gavelin et al., 2021). However, such conventional pairwise meta-analyses on individuals have mixed patients with AD and MCI (Gavelin et al., 2021) or only included CT on individuals with AD (Bahar-Fuchs et al., 2019; Hill et al., 2017) or CS (Cafferata et al., 2021). Furthermore, it is difficult to compare and rank the efficacy of multiple interventions in a pairwise meta-analysis, particularly for combined interventions (i.e., cognitive interventions combined with other non-pharmacological interventions, such as physical exercise). Thus, network meta-analysis extends on the conventional, pairwise meta-analysis by comparing multiple treatments within a network of RCTs to identify the optimal type of cognitive intervention for individuals with AD. Additionally, we aimed to conduct pairwise meta-analyses to evaluate the effects of cognitive interventions on cognition, neuropsychiatric symptoms, depression, quality of life, basic activities of daily living, and instrumental activities of daily living in individuals with AD.

## Method

Our analysis was performed in accordance with the Cochrane Handbook for Systematic Reviews of Interventions (Cumpston et al., 2019) and the Preferred Reporting Items for Systematic Reviews and Meta-analyses (PRISMA) statement (Page et al., 2021a, b).

### Eligibility Criteria

We searched for relevant studies using a population, interventions, comparators, outcomes, and study design (PICOS) approach.

#### Types of Participants

Regarding the population, we included randomized controlled trials (RCTs) of possible or probable AD with a mean age >50 years who were diagnosed using widely recognized diagnostic criteria, including the National Institute of Neurological and Communicative Disorders and Stroke (NINCDS)–Alzheimer’s Disease and Related Disorders Association (ADRDA) and the International Classification of Diseases, Tenth Revision (ICD-10) (Dubois et al., 2007; McKhann et al., 1984). Participants mixed with MCI, where the extent of cognitive impairment or its effects on day-to-day function were insufficient to justify a dementia diagnosis, were not included.

#### Types of Interventions

Regarding the interventions, RCTs involving paper-and-pencil or computerized exercises were included. RCTs involving interventions that targeted a single cognitive domain or multiple cognitive domains were included. CT typically involves guided practice on a set of standardized tasks designed to reflect specific cognitive functions such as memory, attention, or problem-solving. CR aims to directly address those difficulties considered most relevant by the person with dementia and by their family members or supporters, and to target everyday situations in a real-life context. CS encompasses a variety of approaches including reality orientation, validation, or reminiscence. RCTs were also included when the cognitive interventions were combined with other non-pharmacological interventions, such as physical exercise, which were designated “combined interventions”.

#### Types of Controls

Regarding the comparator intervention, active controls (i.e., participants who engaged in a non-structured intervention) and passive controls (i.e., participants on wait lists or standard management) were included.

#### Types of Outcomes

Primary outcomes comprised the change (i.e., from baseline to the end of the treatment) in cognition, including global cognition, confrontation naming (Boston Naming Test), verbal fluency (verbal fluency test), working memory (Digit Span Backward), attention (Digit Span Forward), executive function (Trail Making Test B), immediate and delayed verbal memory (Rey Auditory Verbal Learning Test immediate and delayed recall), immediate and delayed nonverbal memory, processing speed (Trail Making Test A), and visuospatial skills (Clock Drawing Test). Global cognition was evaluated by validated instruments, comprising the Mini-Mental State Examination (MMSE), and the Alzheimer’s Disease Assessment Scale-Cognitive Subscale (ADAS-Cog). Secondary outcomes included neuropsychiatric symptoms (Neuropsychiatric Inventory), depression (Geriatric Depression Scale or Cornell Scale for Depression in Dementia), quality of life (Quality of Life in Alzheimer’s Disease or Dementia-Related Quality of Life), basic activities of daily living (Bayer Activities of Daily Living Scale, Erlangen Test of Activities of Daily Living) and instrumental activities of daily living (Instrumental Activities of Daily Living) (Hill et al., 2017).

### Search Strategy

#### Information Sources

We searched PubMed, Embase, the Cochrane Central Register of Controlled Trials, and Web of Science for RCTs published in English in 2000–2022 August. Earlier studies were excluded, as it is more likely that these studies report outcomes for outdated interventions.

#### Search Strategy

The following medical subject heading (MeSH) terms were used in combination: (dementia OR Alzheimer’s disease) AND (cognitive intervention, cognitive stimulation, cognitive training, cognitive rehabilitation, cognitive method, cognitive therapy, OR cognitive assistance) AND (randomized controlled trial). The full search strategy is shown in Table S1. Additional RCTs from previous reviews and the references of included studies were also considered.

### Data Collection and Analysis

#### Selection Process

Two independent authors screened the titles and abstracts of the included citations and evaluated the full-texts of potentially relevant articles. Consensus was reached by discussion if any disagreement existed.

#### Data Collection Process

Two independent reviewers extracted and verified the relevant data from the included studies, including characteristics of the publications, participants, and interventions, and outcome measures. If disagreements could not be resolved between the two investigators, a consensus was reached by discussion.

#### Data Items

Outcomes were recorded as the mean, standard deviation (SD), and the number of patients who displayed change from baseline. If the change data were not available, the mean, SD and for each treatment group at each time point was extracted. The review authors calculated the required summary statistics from the baseline and post-treatment group means and SD, assuming that the correlation between measurements at baseline and those at the subsequent time points was zero. This method overestimates the SD of the change from baseline, but it is preferable to use a conservative approach in a meta-analysis (Orgeta et al., 2020).

#### Study Risk of Bias Assessment

We used the Grading of Recommendations, Assessment, Development, and Evaluation (GRADE) approach to assess the certainty of the evidence in the included studies on the effect of cognitive interventions compared to control interventions in AD (Guyatt et al., 2011). Risk of bias of each included study was assessed for six domains, including random sequence generation, allocation concealment, blinding of participants, blinding of outcome assessment, incomplete outcome data, and selective outcome reporting.

#### Data Analysis

The analysis was conducted in two steps. First, most parts of the pairwise meta-analyses were conducted using random-effects models in STATA 16.0 software (StataCorp, College Station, TX, USA), while the moderator analysis and forest plots of relative treatment effects were conducted to investigate the potential sources of heterogeneity using the statistical package *metafor* (3.4-0) and *forestplot* (2.0.1) in the R software (4.1.2). To adjust for bias resulting from small sample sizes, the effect size for continuous outcomes was calculated as the standardized mean difference (SMD absolute values of <0.30, 0.30–0.60, and >0.80 indicate small, moderate, and large effects, respectively) with 95% confidence intervals (CIs). (Higgins et al., 2003). We pooled the Hedges’ g to correct the effect size for small sample sizes (Hedges & Olkin, 1985).

A random-effects network meta-analysis of cognitive outcomes using MMSE and ADAS-cog was conducted to compare the four cognitive intervention types by using a Bayesian statistical model, and forming a connected network that integrated both direct and indirect evidence using STATA software network (Caldwell et al., 2005). Only one included study used the Montreal Cognitive Assessment, which prevented further analysis on outcomes from this test. We used the Markov chain Monte Carlo method to conduct the network meta-analysis, involving non-informative prior to distributions (Mavridis & Salanti, 2013). Our model generated 50000 iterations, and the first 5000 were discarded as burn-in. We ranked the four interventions according to the surface under the cumulative ranking curve (SUCRA) of the efficacy of different cognitive interventions. Higher SUCRA values indicate that an intervention is more likely to be highly effective, while values closer to zero indicate that the intervention is more likely to be in the bottom rank (Salanti et al., 2011).

#### Moderator Analysis and Investigation of Heterogeneity

Study heterogeneity was assessed using the *I*^2^ and τ^2^ statistics (*I*^2^ of 25%, 50%, and 75% indicate mild, moderate, and high levels of heterogeneity, respectively) (Higgins et al., 2003). As no guideline for interpretation of τ^2^ exists in literature, we selected a cutoff point of 0.10 based on a previous empirical study (Morze et al., 2022). Moderator analyses were performed using cognitive intervention type (CS, CT, CR, or combined interventions), setting (individual- or group-based intervention), region (Asia or Europe plus America), and control group type (active or passive) to investigate the potential sources of heterogeneity.

#### Assessment of Statistical Inconsistency

The inconsistency between direct and indirect approaches throughout the network was assessed using the node-splitting approach (van Valkenhoef et al., 2016), with *p* < 0.05 indicating the presence of inconsistency (Higgins et al., 2012).

#### Sensitivity Analysis and Reporting Bias Assessment

A sensitivity analysis was used to assess the stability of meta-analysis results. Egger’s test and funnel plots were used to assess publication bias among the included studies (Sterne et al., 2011).

### Summary of Findings and Assessment of Certainty of Evidence

The GRADE approach was used to assess the certainty of evidence for the included studies reporting on the treatment effect of cognitive intervention in AD compared to a control condition. Risk of bias, imprecision, inconsistency, indirectness, and publication bias were the domains used to rate the overall certainty. 

## Results

### Study Selection

**Fig. 1:**
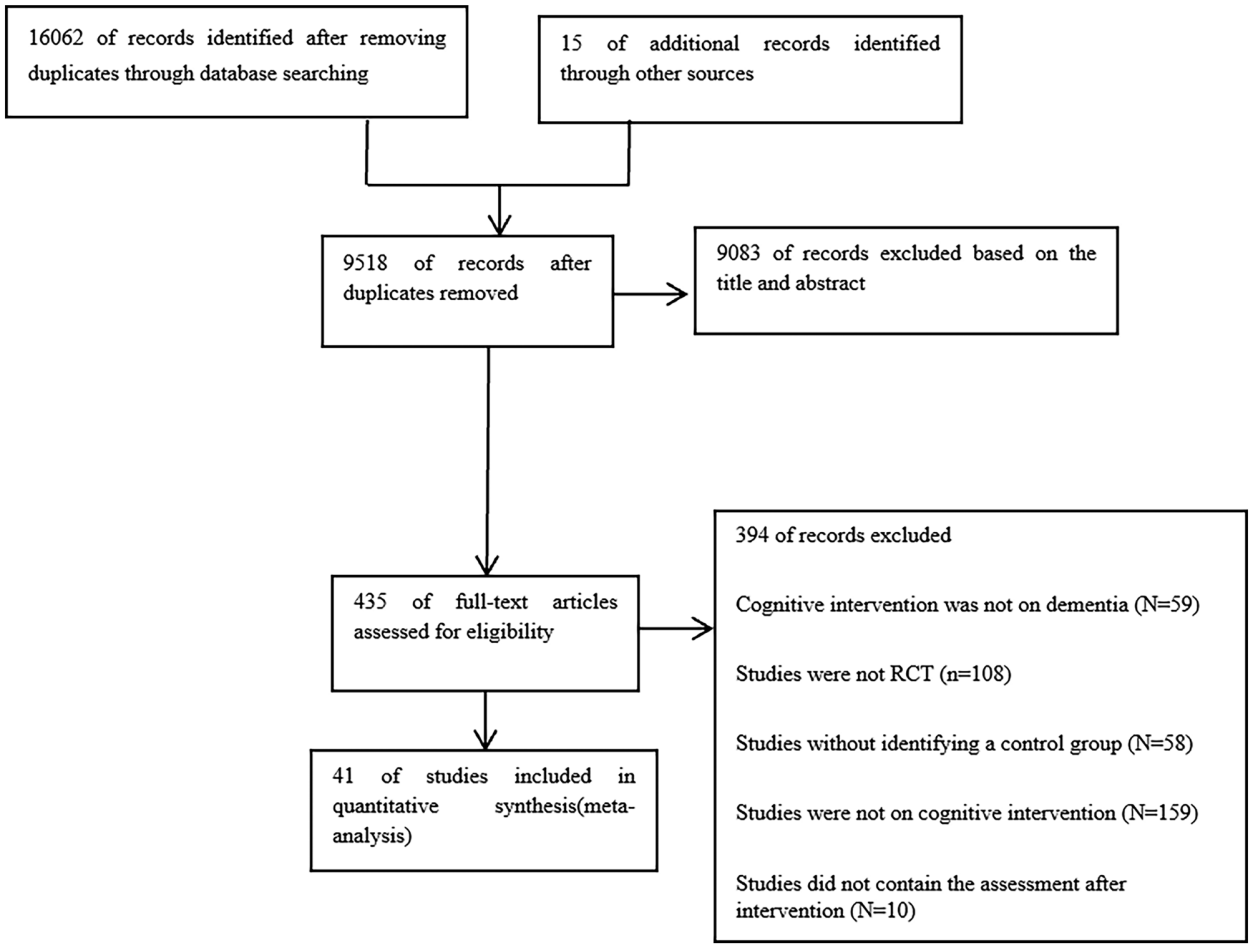
Flowchart of article selection

A total of 9518 records from the database search were retained after removing duplicates. Of these records, 9083 were excluded based on titles and abstract screening. We assessed 435 full-text articles for eligibility and 394 of records were excluded. Finally, we found that 41 studies were eligible for inclusion. There were no disagreements between the two independent reviewers regarding the selection of studies. A flowchart of the included studies is shown in Fig. 1.

### Participant Characteristics and Quality of Included Studies

The 41 included studies involved 2179 individuals with AD, comprising 1103 individuals in intervention groups and 1076 individuals in control groups. The mean age ranged from 68.67 to 88.25, and the percentage of females ranged from 22.5% to 82.5%. The demographic characteristics of the participants in the included studies are shown in Table 1. The quality (based on the GRADE approach) of each included study is presented in Figs. S1 and S2. Overall, most of included studies did not provide concrete information about random sequence generation, allocation concealment, and blinding. Therefore, the quality of the included RCTs was considered to be only moderate, overall.

**Table 1:** Characteristics of Included Studies

Intervention	Author/year	Duration		Control	Mean age		Sex(%F emale)	Region
					Exp	Con		
CS	Davis et al., 2001	60min, once a week, 5weeks,5h total	*Individual*	AC	68.67	72.56	56.8%	USA
	Spector et al., 2003	45min, twice a week, 7 weeks, 10.5h total	*In groups*	PC	85.7	84.7	80.6%	England
	Wang 2007	60min, once a week, 8weeks, 8h total	*In groups*	PC	79.76	78.92	51.0%	Taiwan
	Niu et al., 2010	45min, twice a week, 10weeks, 15h total	*Individual*	AC	80.56	79.13	21.9%	China
	Coen et al., 2011	45min, twice a week, 7 weeks	*In groups*	PC	78.4	81.3	51.9%	Ireland
	Lee et al., 2013	30min, twice a week, 12h total	*Individual*	PC	NA	NA	41.6%	Hong Kong
	Yamanaka et al., 2013	45 min, twice a week, 7 weeks, 10.5h total	*In groups*	PC	84.12	83.73	78.6%	Japan
	Mapelli et al., 2013	60min, five times per week, 8 weeks, 40htotal	*Individual*	PC	82.6	84.7	NA	Italy
	Cove et al., 2014	45 min,once a week, 14 weeks, 10.5h total	*Individual*	PC	76.8	77.8	46.8%	UK
	Orrell et al., 2014	45 min, twice a week, 7 weeks, 10.5h total	*In groups*	PC	82.7	83.5	75%	UK
	Capotosto et al., 2017	45min, twice a week,7 weeks, 10.5h total	*In groups*	AC	88.25	86.52	69.2%	Italy
	López et al., 2020	60min, three times a week, 24 week, 72h total	*Individual*	AC	83.3	80.5	75%	Spain
	Oliveira et al., 2021	45min, twice a week, 5 weeks, 7.5h total	*In groups*	PC	83.24		70.59%	Portugal
	Justo-Henriques et al., 2022	45min, once a week, 47 weeks, 35h total	*Individual*	PC	78.53	79.21	61.0%	USA
CT	Jelcic et al., 2012	60min, twice a week, 12 weeks, 24h total	*Individual*	AC	82.9	81.8	82.5%	Italy
	Jelcic et al., 2014			AC	82.7	82.3	80%	Italy
	Bergamaschi et al., 2013	120min, five times per week,20 weeks, 200h total	*In groups*	AC	78.19	77.72	NA	Italy
	Huntley et al., 2017	30min, 18seesions, 8 weeks, 9h total	*Individual*	AC	79.4	80.13	NA	England
	Venturelli et al., 2016	60min, five times a week, 12 weeks, 60h total	*Individual*	PC	86	84	72.5%	Italy
	Giuli et al., 2016	45min, once a week, 10 weeks, 7.5h total	*Individual*	AC	76.5	78.7	66.3%	Italy
	Giovagnoli et al., 2017	45min a day, twice a week,12 weeks,18h total	*In groups*	AC	71.69	75.31	69.2%	Italy
	Trebbastoni et al., 2018	75min a day, twice a week, 24 weeks,60h total	*In groups*	PC	74.26	76.01	60%	Italy
	Fonte et al., 2019	90 min a day, three times a week, 24 weeks, 10 total	*In groups*	PC	79	80	65%	Italy
	Kang et al., 2019	60min, twice a week, 12 weeks,24h total	*Individual*	PC	69.1	68.9	22.5%	Korea
	Casoli et al., 2020	45min, once a week, 10 weeks, 7.5h total	*Individual*	AC	76.32	78.74	62.7%	Italy
	Shyu, 2022	30min, once a week, 6 weeks,3h total	*Individual*	PC	82	80	37%	Taiwan
CR	Bottino et al., 2005	90min, once a week, 5 month, 30h total	*Individual*	PC	74.67	72.86	69.2%	Brazil
	Brueggen et al., 2017	60min, once a week, 12 weeks, 12h total	*In groups*	PC	70.57	69.75	53.3%	Germany
	Kurth, 2021	60min, once a week, 12 weeks, 12h total	*Individual*	AC	72.4	74.9	34%	Belgium
Combined	Tarraga et al., 2006	20min, three times per week, 24 weeks, 24h total(multimedia) 8h(CS)	*Individual*	PC	75.8	76.9	81.5%	Spain
	Buschert et al., 2011	120 min,once a week, 48h total(CS+CT)	*In groups*	AC	77.3	74.2	46.7%	Germany
	Maci et al., 2012	60min, five times a week, 60h total(CS) 60min, five times a week, 60h total(physical exercise)	*In groups*	PC	75	70.3	57.2%	Italy
	Luttenberger et al., 2012	30min, seven times a week, 183h total(CS) 30min, seven times a week, 183h total(physical exercise)	*In groups*	AC	84.1	84.64	78.8%	Germany
	Kim, 2016	120min, once a week, 24 weeks, 48h total(CS) 120min, once a week, 24 weeks, 48h total(physical exercise)	*Individual*	PC	78.44	78.52	69.9%	Korea
	Tokuchi, 2016	60-120min, once or twice a week,10h total(CR combined with physical exercise)	*In groups*	PC	79.0	78.8	65.1%	Japan
	Fernández-Calvo et al., 2015	90 min a day, three times a week, 16 weeks,72h total	*Individual*	PC	74.32	72.33	58.18%	Spain
	Okamura et al., 2018	5min, once or more a week, 24 weeks, 2h at least(CT combined with exercise)	*Individual*	AC	82.4	79.2	70%	Japan
	Young et al., 2019	60min, twice a week, 7 week, 14h total(CS) 15min per times(physical exercise)	*In groups*	PC	80.53	79.86	80.2%	Hong Kong
	Kim 2020	60min, five times per week, 24h total(CS combined with exercise)	*Individual*	PC	80.6	77.88	74.29%	Korea
	Sado et al., 2020	30 min, five times per week,120h total(CS+CT)	*In groups*	PC	83.9	86.3	73.3%	Japan
	Tanaka, 2017	45min, twice a week, 8 weeks(CT or CS + physical exercise)	*In groups*	PC	84.2	88.1	58.1%	Japan

### Effects of Intervention

The summary of findings showing the pooled data for the main comparison of cognitive intervention groups versus controls is shown in Table S2.

#### Global Cognition

**Fig. 2:**
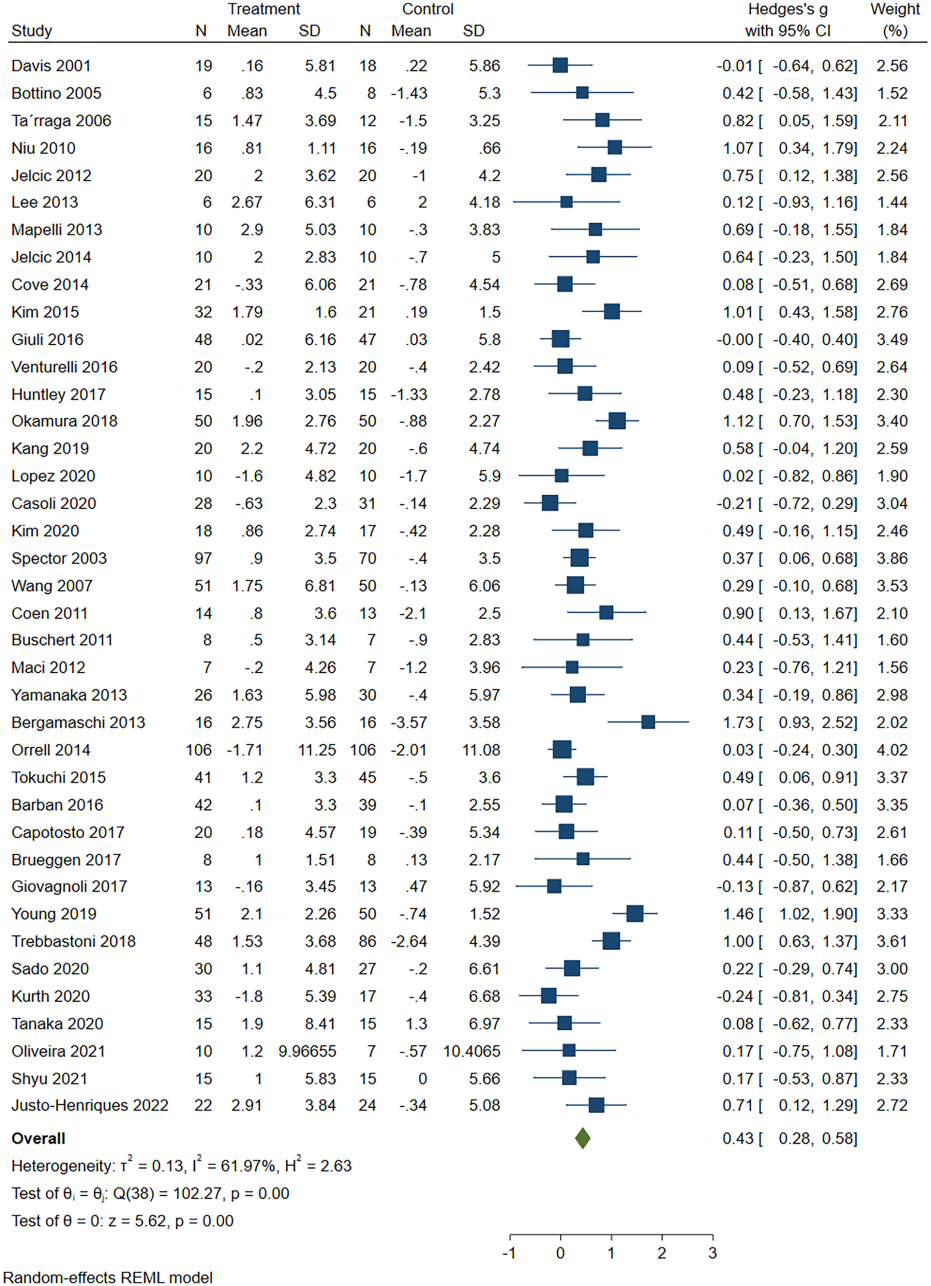
Effect of cognitive intervention on global cognitive functions using MMSE, Mini-Mental State Examination

**Fig. 3:**
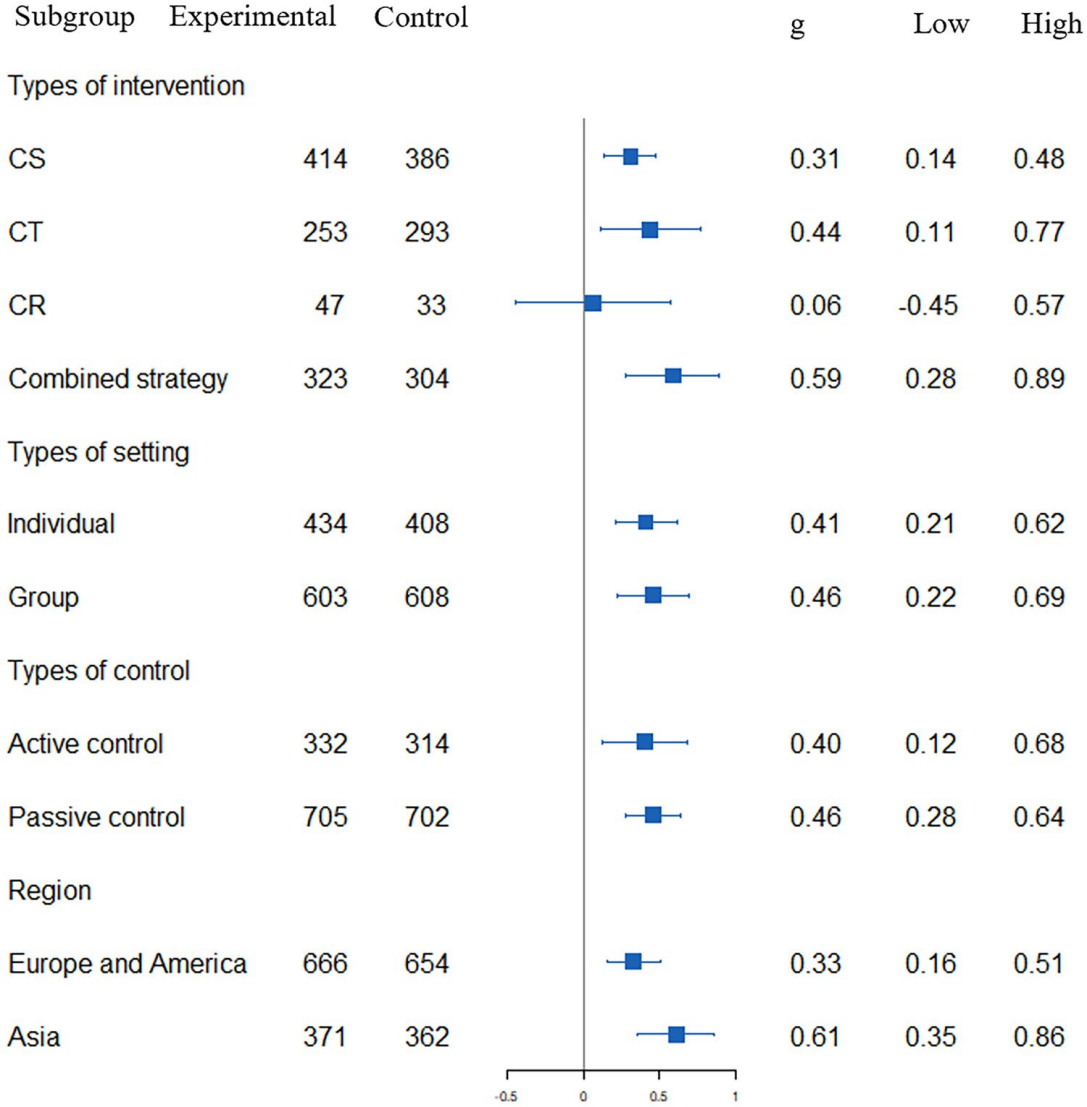
The moderator analysis of cognitive outcome based on *MMSE* Mini-Mental State Examination, *CS* cognitive stimulation, *CT* cognitive training, *CR* cognitive rehabilitation

There was a moderate and significant post-intervention improvement in MMSE outcomes for the cognitive intervention groups compared to the controls (39 studies, g = 0.43, 95% CI: 0.28 to 0.58, *p* < 0.01; Q = 102.27, df = 38, *p* < 0.01; *I*^2^ =61.97%, τ^2^=0.13, Fig. 2). The funnel plot and Egger’s test (*p* = 0.872) did not reveal any evidence of publication bias (Fig. 3).

There was a moderate and significant post-intervention improvement in ADAS-Cog outcomes for the cognitive intervention groups compared to the controls (nine studies, g = -0.33, 95% CI: -0.53 to -0.12, *p* < 0.01; Q = 7.59, df = 8, *p* = 0.47; I^2^ = 0.01%, τ^2^ = 0.00, Fig. S3). The funnel plot and Egger’s test *(p =* 0.448) did not reveal any evidence of publication bias.

#### Moderator Analyses

Moderator analyses were conducted to determine whether within-group treatment efficacy varied as a function of participant and study characteristics. Specifically, four moderator variables were examined, including cognitive intervention type (CS, CT, CR, or combined therapy), setting (individual- or group-based intervention), region (Asia or Europe plus America), and control group type (active or passive).

##### Cognitive Intervention Type

The moderator analysis revealed a significant improvement for global cognition in CT (11 studies, g = 0.44, SE = 0.17, 95% CI:0.11 to 0.77, z = 2.63,* p* < 0.01), CS (14 studies, g = 0.31, SE = 0.08, 95% CI: 0.14 to 0.48, z = 3.58, *p* < 0.01), combined therapy (11 studies, g = 0.59, SE = 0.16, 95% CI: 0.28 to 0.89, z = 3.77,* p* < 0.01), but not for CR (three studies, g = 0.06, SE = 0.26, 95% CI: -0.45 to 0.57, z = 0.24, *p* = 0.81). The degree of heterogeneity within the groups was significant (Q_within_ = 86.55, df = 35,* p* < 0.01) and the degree of heterogeneity between the groups was non-significant (Q_between_ = 2.83, df = 3, *p* = 0.41).

##### Setting

The moderator analysis revealed a significant improvement for global cognition in individual-based (21 studies, g = 0.41, SE = 0.10, 95% CI:0.21 to 0.62, z = 3.95, *p* < 0.01) and group-based (18 studies, g = 0.46, SE = 0.12, 95% CI: 0.22 to 0.69, z = 3.81, *p* < 0.01). The degree of heterogeneity within the groups was significant (Q_within_ = 99.77, df = 37, *p* < 0.01), and the degree of heterogeneity between the groups was non-significant (Q_between_ = 0.07, df = 1,* p*=0.78).

##### Region

The moderator analysis revealed a significant improvement for global cognition in Asia (13 studies, g = 0.61, SE = 0.13, 95% CI: 0.35 to 0.86, z = 4.65, *p* < 0.01) and in Europe combined with America (26 studies, g = 0.33, SE = 0.09, 95% CI: 0.16 to 0.51, z = 3.67, *p* < 0.01). The degree of heterogeneity was significant both within the groups (Q_within_ =86.33, df = 37, *p* < 0.01) and between the groups (Q_between_ = 3.14, df = 1,* p* = 0.08).

##### Control Group Type

The moderator analysis revealed a significant improvement in global cognition when compared with r passive controls (23 studies, g = 0.46, SE = 0.09, 95% CI: 0.28 to 0.64, z = 4.97, *p* < 0.01) and when compared with active controls (16 studies, g = 0.40, SE = 0.14, 95% CI: 0.12 to 0.68, z = 2.81, *p* < 0.01). The degree of heterogeneity within the groups was significant (Q_within_ = 99.45, df = 37,* p* < 0.01), and the degree of heterogeneity between the groups was non-significant (Q_between_ = 0.16, df = 1,* p* = 0.69).

##### Network Meta-Analysis

**Fig. 4:**
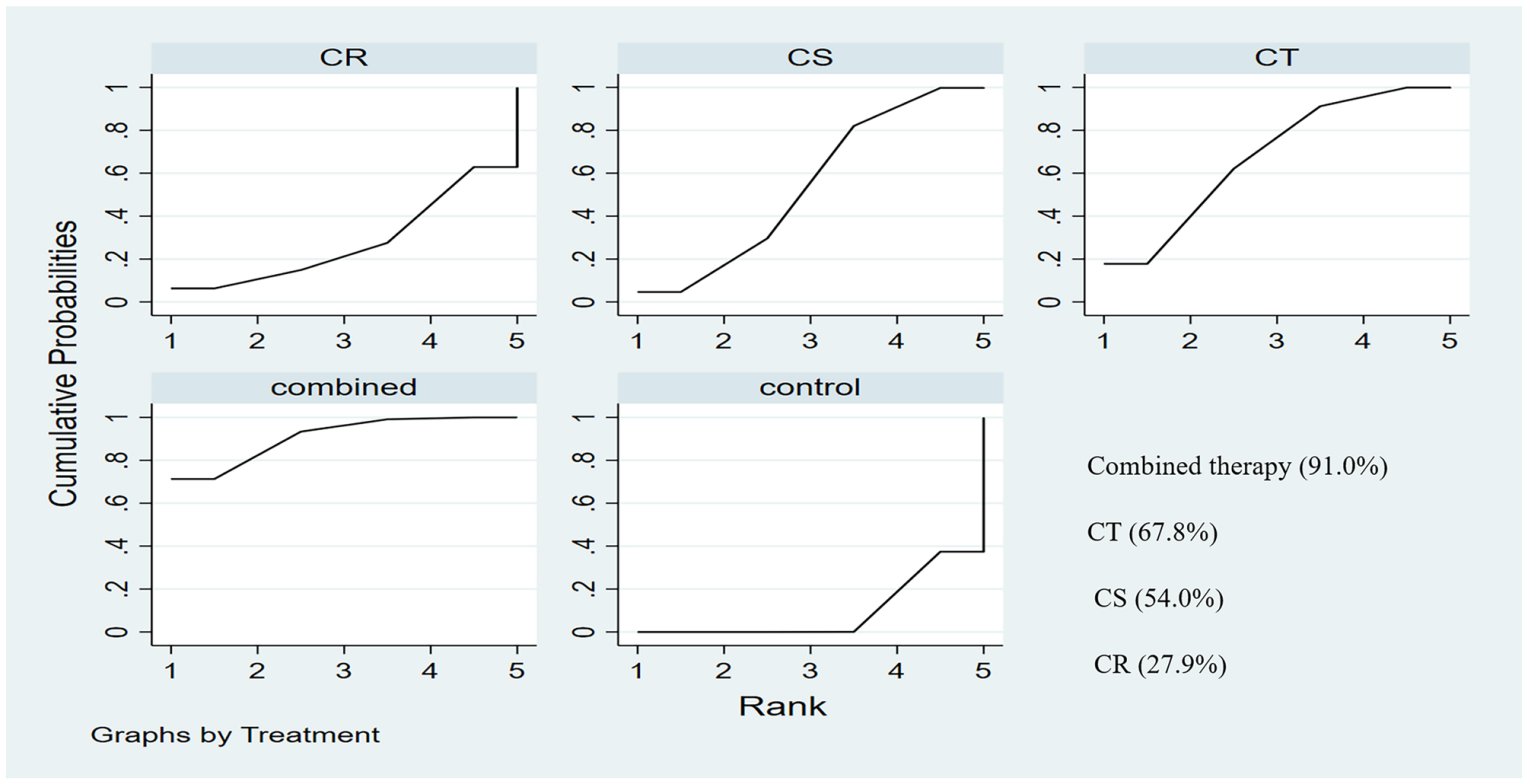
The rankings of overall cognitive interventions based on SUCRA line

We also conducted a network meta-analysis to rank the efficacy of the cognitive intervention types (Fig. S4). Among the 39 studies that used the MMSE, there were 14 studies on CS (Capotosto et al., 2017; Coen et al., 2011; Cove et al., 2014; Davis et al., 2001; Justo-Henriques et al., 2022; Lee et al., 2013; Lopez et al., 2020; Mapelli et al., 2013; Niu et al., 2010; Oliveira et al., 2021; Orrell et al., 2014; Spector et al., 2003; Wang, 2007; Yamanaka et al., 2013), 11 studies on CT (Bergamaschi et al., 2013; Casoli et al., 2020; Giovagnoli et al., 2017; Giuli et al., 2016; Huntley et al., 2017; Jelcic et al., 2014; Jelcic et al., 2012; Kang et al., 2019; Shyu et al., 2022; Trebbastoni et al., 2018; Venturelli et al., 2016), three studies on CR (Bottino et al., 2005; Brueggen et al., 2017; Kurth et al., 2021), and 11 studies on combined interventions (Barban et al., 2016; Buschert et al., 2011; Kim, 2020; Kim et al., 2016; Maci et al., 2012; Okamura et al., 2018; Sado et al., 2020; Tanaka et al., 2021; Tarraga et al., 2006; Tokuchi et al., 2016; Young et al., 2019). The SUCRA value was used to rank the efficacy of each intervention (Fig. 4). Higher SUCRA values indicate a higher likelihood that a treatment is in the top rank or is highly effective, while zero represents a higher likelihood that a treatment is in the bottom rank. Combined interventions had the highest SUCRA value (90.7%), followed by CT (67.8%), CS (53.4%), and lastly CR (28.9%). The inconsistency test based on network analysis revealed no significant global inconsistency (*p* = 0.965), and the node-splitting approach revealed that relatively reliable evidence can be drawn from the absence of statistical inconsistency (*p >* 0.05; CS versus controls, *p* = 0.803; CS versus combined intervention strategies, *p* = 0.869; combined intervention strategies versus controls, *p* = 0.968). Pairwise comparisons of all cognitive interventions are presented in the network league table displayed in Table S3.

Among nine studies on ADAS-Cog, there were two studies on CT (Fonte et al., 2019; Giuli et al., 2016), three studies on combined therapy (Fernandez-Calvo et al., 2015; Luttenberger et al., 2012; Tarraga et al., 2006), three studies on CS (Capotosto et al., 2017; Coen et al., 2011; Lopez et al., 2020), and one study on CR (Bottino et al., 2005) (Fig. S5). The SUCRA value was used to rank the efficacy of each intervention (Fig. S6). CT had the highest SUCRA value (79.6%), followed by combined therapy (72.3%), CR (55.5%), and lastly CS (18.6%). However, owing to the limited studies that used the ADAS-Cog, no comparison was available to assess statistical consistency, which may influence the reliability of results.

#### Specific Cognitive Domains

##### Working Memory

The effect size for working memory was moderate and significant (seven studies, g = 0.36, 95% CI: 0.11 to 0.61,* p* = 0.01; Q = 3.62, df = 6, *p* = 0.73; I^2^ = 0%, τ^2^ = 0.00). The funnel plot and Egger’s test (*p* = 0.7406) did not reveal publication bias (Fig. S7).

##### Verbal Memory

The effect size for immediate verbal memory was moderate and significant (five studies, g = 0.37, 95% CI: 0.12 to 0.62, *p* < 0.01; Q = 1.62, df = 4, *p* = 0.81; I^2^ = 0.0%, τ^2^ = 0.00). The funnel plot and Egger’s test (*p* = 0.35) did not reveal publication bias (Fig. S7).

The effect size for delayed verbal memory was small and significant (five studies, g = 0.26, 95% CI: 0.03 to 0.49, *p* = 0.03; Q = 1.86, df = 4, *p* = 0.76; I^2^ = 0.0%, τ^2^ = 0.00). The funnel plot and Egger’s test (*p* = 0.412) did not reveal publication bias (Fig. S7).

##### Verbal Fluency

The effect size for verbal fluency was small and significant (seven studies, g = 0.26, 95% CI: 0.05 to 0.47, *p* = 0.02; Q = 6.47, df = 6, *p* = 0.37; I^2^ = 0.0%, τ^2^ = 0.00). The funnel plot and Egger’s test (*p* = 0.200) did not reveal publication bias (Fig. S7).

##### Confrontation Naming

The effect size for confrontation naming was moderate and significant (seven studies, g = 0.42, 95% CI: 0.18 to 0.66, *p*<0.01; Q = 4.99, df = 6, *p* = 0.55; I^2^ = 0.0%, τ ^2^ =0.00). The funnel plot and Egger’s test (*p* = 0.330) did not reveal publication bias (Fig. S7).

##### Attention

The effect size for attention was moderate and significant (six studies, g = 0.32, 95% CI: 0.03 to 0.62, *p* = 0.03; Q = 4.30, df = 5, *p* = 0.51; I^2^ = 0.0%, τ^2^ = 0.00). The funnel plot and Egger’s test (*p* = 0.701) did not reveal publication bias (Fig. S7).

##### Other Specific Cognitive Domains

**Fig. 5:**
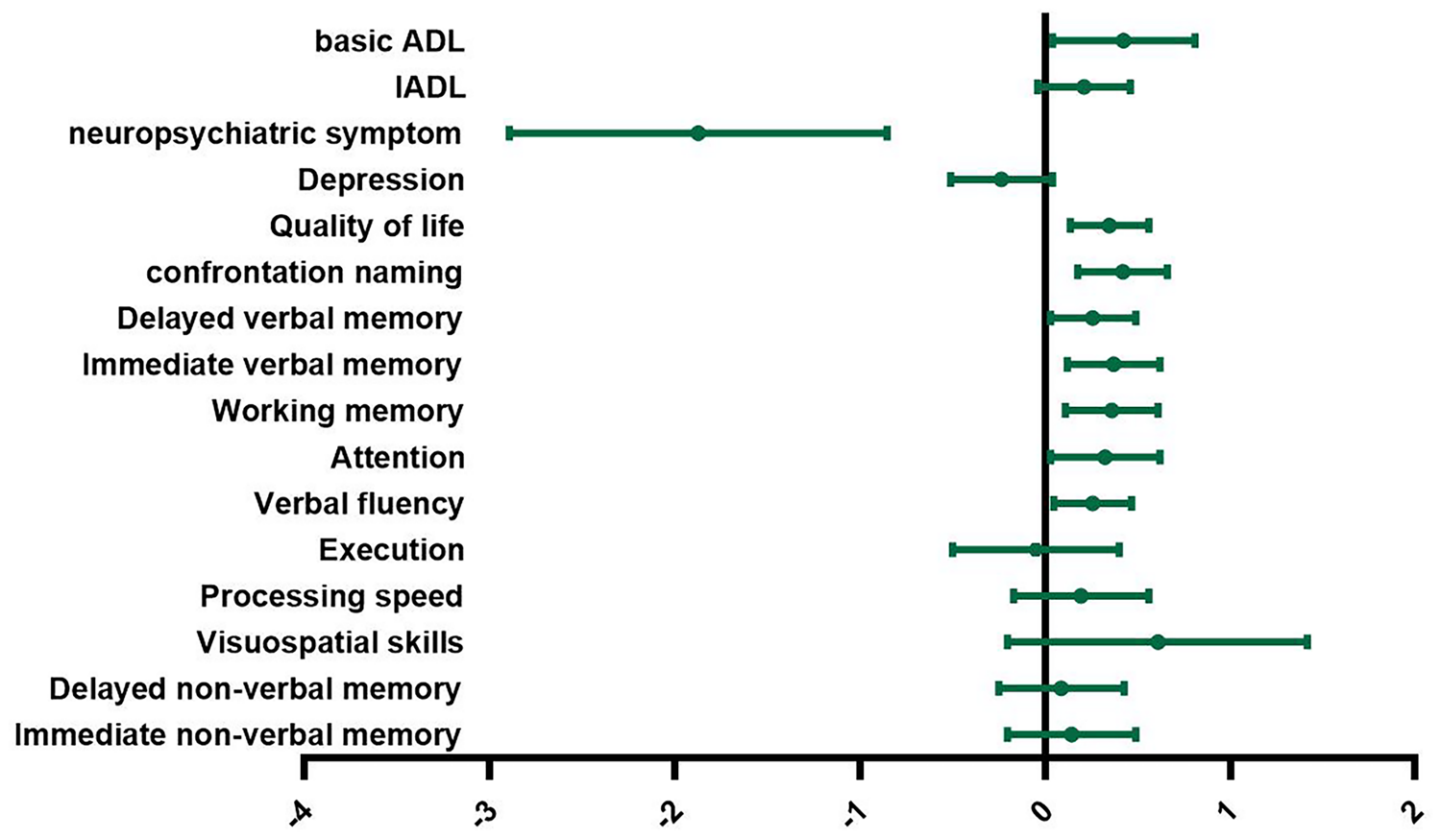
Efficacy of cognitive intervention in Alzheimer’s disease within individual domains, *basic ADL basic activities of daily living*, *IADL* Instrumental activities of daily living

Non-significant results were found for executive function (four studies, g= –0.05, 95% CI: –0.50 to 0.40, *p* = 0.82; Q = 2.30, df = 3, *p* = 0.51; I^2^ = 0.0%,τ^2^ = 0.07), visuospatial skills (three studies, g = 0.61, 95% CI: –0.20 to 1.42, *p* = 0.14; Q = 6.99, df = 2, *p* = 0.03; I^2^ = 72.22%, τ^2^ = 0.36), processing speed (six studies, g = 0.20, 95% CI: –0.17 to 0.56, *p* = 0.29; Q = 6.70, df = 5, *p* = 0.24; I^2^ = 35.52%, τ^2^ = 0.07, immediate nonverbal memory (four studies, g = 0.15, 95% CI: –0.20 to 0.49, *p* = 0.40; Q = 0.48, df = 3, *p* = 0.92; I^2^ = 0.0%, τ^2^ = 0.00) and delayed nonverbal memory (four studies, g = 0.09, 95% CI: –0.25 to 0.43,* p* = 0.61; Q = 0.22, df = 3, *p* = 0.97; I^2^ = 0.0%,τ^2^ = 0.00). The results for specific cognitive domains are summarized in Fig. 5.

##### Subgroup Analyses

Interestingly, there were contradictory results regarding the effects of cognitive intervention type on working memory, attention, and confrontation naming. CT improved these specific cognitive domains, while CS did not improve working memory or attention, and CR did not improve confrontation naming. There were not enough studies for subgroup analyses for combined interventions.

#### Non-cognitive Domains

##### Neuropsychiatric Symptoms and Depression

The effect size for neuropsychiatric symptoms was large and significant (four studies, g = –1.87, 95% CI: –2.89 to –0.85, *p* < 0.01; Q = 33.28, df = 3, *p* < 0.01; I^2^ = 89.83%, τ^2^ = 0.97). The funnel plot and Egger’s test did not reveal publication bias (*p =* 0.868) (Fig. S8).

No significant results were found for depression (12 studies, g = –0.24, 95% CI: –0.51 to 0.04, I^2^ = 61.27%). The funnel plot and Egger’s test did not reveal significant publication bias (*p* = 0.572).

##### Quality of Life and Activities of Daily Living

The effect size for quality of life was moderate and significant (six studies, g = 0.35, 95% CI: 0.14 to 0.56, *p* < 0.01; Q = 2.91, df = 5, *p* = 0.71; I^2^ = 0.0%, τ^2^ = 0.00). The funnel plot and Egger’s test (*p* = 0.948) did not reveal publication bias (Fig. S9).

The effect size for basic activities of daily living was moderate and significant (four studies, g=0.42, 95% CI: 0.04 to 0.81, *p* = 0.03; Q = 8.04, df = 3, *p* = 0.05; I^2^ = 60.82%, τ^2^ = 0.09). The funnel plot and Egger’s test (*p* = 0.903) did not reveal publication bias (Fig. S10).

No significant results were found for instrumental activities of daily living (five studies, g = 0.21, 95% CI: –0.04 to 0.46, *p* = 0.10; Q = 0.74, df = 4, *p* = 0.95; I^2^ = 0.0%, τ^2^ = 0.00). These results are summarized in Fig. 5.

##### Subgroup Analyses

Interestingly, there were contradictory results regarding the effects of different cognitive intervention types on depression. Combined interventions improved depression, while CS and CT did not improve depression. There were not enough studies for subgroup analyses of CR.

## Discussion

To the best of our knowledge, this is the first meta-analysis to analyze the effects of cognitive intervention types on cognition, neuropsychiatric symptoms, depression, quality of life, basic activities of daily living, and instrumental activities of daily living in individuals with AD. Based on the results of 41 RCTs of moderate quality, we conclude that cognitive interventions are a viable approach to improve cognition in AD, and that the optimal approach is to combine interventions (i.e., cognitive interventions combined with other non-pharmacological interventions; SUCRA = 90.7%). Our robust results show that cognitive interventions, and in particular, CT can benefit global cognition (more specifically, working, verbal memory, attention, confrontation naming for moderate confidence, and verbal fluency for low confidence), neuropsychiatric symptoms, basic activities of daily living for low confidence, and quality of life, with moderate confidence.

Based on this review, the applicability of CT, characterized by standard tasks for improving specific cognitive functions, is much broader than that of CS and CR, potentially because CT may increase functional connectivity in the medial temporal lobe and cause topological changes in the anterior cingulum in individuals with AD (Barban et al., 2017). Importantly, CT combined with other non-pharmacological interventions, including physical exercise, can influence brain plasticity through distinct and complementary paths (Bherer, 2015). A recent study found that simultaneous rather than sequential training might be better to achieve maximal benefit (Gavelin et al., 2021). The results for CR were the poorest among the four cognitive intervention types, and there were a limited number of studies on CR compared to CT and CS. We conclude that combined interventions might be the most beneficial approach for individuals with AD, while CR might not be the best option.

The moderate effect sizes for most memory and language outcomes are very promising, as memory and language issues are highly common in AD. Interestingly, although there was a moderate effect for working memory, there was a non-significant effect for executive function, which is a key predictor of functional decline (Lacreuse et al., 2020). This is consistent with previous meta-analyses results regarding CT in AD (Bahar-Fuchs et al., 2019) and CT in MCI (Hill et al., 2017). A previous study found that executive function training supported brain functioning in individuals who were starting to experience cognitive decline (Cheng, 2016). Thus, we believe that more research on executive function training is needed.

Depression is common in individuals with cognitive impairment (Ismail et al., 2017). Previous studies found moderate effect sizes regarding the effects of cognitive interventions on depression in individuals with MCI (Sherman et al., 2017). However, in agreement with our findings (i.e., no significant results for depression), another study showed that cognitive intervention failed to improve depression in AD (Hill et al., 2017). Depression can increase the risk of progression to dementia in individuals with MCI (Baruch et al., 2019). Thus, if cognitive intervention improved depression in the early stage, progression to dementia may be reduced. For individuals with AD, subjective measures of depression and instrumental activities of daily living might be limited.

Although we performed a comprehensive literature search and fully analyzed the resultant data, our meta-analysis has several limitations. First, there was no or low study heterogeneity for all outcomes, though as only subgroup analysis for combined interventions had high heterogeneity, the reliability of results may have been less affected. Besides, the limited number of studies might influence the inconsistency between the direct and indirect comparisons, especially those which compared the efficacy of the different approaches to cognitive intervention, and thus we believe more data are needed to directly compare the efficacy between different interventions. Moreover, although the results of Egger’ test suggested a low possibility of publication bias, it cannot be concluded that there is no funnel asymmetry since a limited number of studies were included for several meta-analyses. Meanwhile, as in most published meta-analyses, the literature search was limited to English-language articles. Lastly, most of the RCTs concentrated on short-term cognitive outcomes, so we lacked sufficient data to evaluate the clinical efficacy of long-term cognitive interventions, and to evaluate whether the effects are maintained in the long-term after the interventions are completed.

## Conclusion

Our findings suggest that cognitive interventions can improve cognition, neuropsychiatric symptoms, basic activities of daily living, and quality of life in individuals with AD. Combined intervention was the most effective cognitive intervention type, followed by CT, CS, and CR. However, the meta-analysis was limited by the fact that long-term effects were not reported. We believe that long-term follow-up and large samples are needed to further investigate the effects of cognitive interventions on these functions.

### Supplementary Information

Below is the link to the electronic supplementary material.

Supplementary file1 (DOCX 14 KB)

Supplementary file2 (DOCX 21 KB)

Supplementary file3 (DOCX 17 KB)

Supplementary file4 (DOC 98 KB)

Supplementary file5 (DOCX 14 KB)

Supplementary file6 (DOC 16 KB)

Supplementary file7 (DOC 126 KB)

Supplementary file8 (DOC 410 KB)

Supplementary file9 (DOC 254 KB)

Supplementary file10 (DOC 119 KB)

Supplementary file11 (DOC 267 KB)

Supplementary file12 (DOC 323 KB)

Supplementary file13 (DOCX 600 KB)

Supplementary file14 (DOCX 21 KB)

Supplementary file15 (DOC 272 KB)

Supplementary file16 (DOCX 244 KB)

Supplementary file17 (DOCX 17 KB)

Supplementary file18 (DOCX 18 KB)

Supplementary file19 (DOCX 18 KB)

Supplementary file20 (DOCX 244 KB)

## Data Availability

The data of original data was available in the Github (https://github.com/xiangchunchen2012/meta/tree/data/data) and computer code are available in the Github (https://github.com/xiangchunchen2012/meta/tree/data).
