# Correction to: Comparison of Cognitive Intervention Strategies for Individuals with Alzheimer’s Disease: A Systematic Review and Network Meta-analysis

**DOI:** 10.1007/s11065-023-09591-6

**Published:** 2023-04-10

**Authors:** Chunchen Xiang, Yumei Zhang

**Affiliations:** 1 Department of Neurology, Beijing Tiantan Hospital, Capital Medical University, Beijing, China; 2 Department of Rehabilitation Medicine, Beijing Tiantan Hospital, Capital Medical University, Beijing, 100070 China; 3 Center of Stroke, Beijing Key Laboratory of Translational Medicine for Cerebrovascular Disease, Beijing Institute for Brain Disorders, Beijing, China; 4 China National Clinical Research Center for Neurological Diseases, Beijing, China

**Correction to: Neuropsychology Review**

10.1007/s11065-023-09584-5

The article “Comparison of Cognitive Intervention Strategies for Individuals With Alzheimer’s Disease: A Systematic Review and Network Meta‑analysis”, written by Chunchen Xiang and Yumei Zhang, was originally published electronically on the publisher’s internet portal on Marc 16, 2023 without open access. With the author(s)’ decision to opt for Open Choice the copyright of the article changed on March 16, 2023 to © The Authors, 2023 and the article is forthwith distributed under a Creative Commons Attribution 4.0 International License, which permits use, sharing, adaptation, distribution and reproduction in any medium or format, as long as you give appropriate credit to the original author(s) and the source, provide a link to the Creative Commons licence, and indicate if changes were made.

The images or other third party material in this article are included in the article?s Creative Commons licence, unless indicated otherwise in a credit line to the material. If material is not included in the article?s Creative Commons licence and your intended use is not permitted by statutory regulation or exceeds the permitted use, you will need to obtain permission directly from the copyright holder.

To view a copy of this licence, visit http://creativecommons.org/licenses/by/4.0/.

The original article has been corrected.

