# Supplementary material for: Comparison of Cognitive Intervention Strategies for Individuals With Alzheimer’s Disease: A Systematic Review and Network Meta-analysis
Source: Neuropsychol Rev. 2023 Mar 16;34(2):402–16. doi: 10.1007/s11065-023-09584-5 (PMC11166762; doi:10.1007/s11065-023-09584-5)
Supplement: Supplementary file 1 — Supplementary file1 (DOCX 14 KB) [file 11065_2023_9584_MOESM1_ESM.docx]

#1. "alzheimer disease"[MeSH Terms] OR ("alzheimer"[Title/abstract] AND "disease" Title/abstract]) OR "dementia"[ Title/abstract]

#2. cognitive intervention [Title/abstract] OR cognition-based intervention [Title/abstract] OR cognition-focused intervention [Title/abstract] OR cognitive support [Title/abstract] OR cognitive training [Title/abstract] OR computer-based cognitive training [Title/abstract] OR computerized cognitive training [Title/abstract] OR attention training [Title/abstract] OR attentional training [Title/abstract] OR executive training [Title/abstract] OR memory training [Title/abstract] OR language training [Title/abstract] OR visuospatial training [Title/abstract] OR orientation training [Title/abstract] OR cognitive stimulation [Title/abstract] OR cognitive rehabilitation [Title/abstract] OR cognitive therapy [Title/abstract] OR ADL training [Title/abstract] OR activity of daily training [Title/abstract]

#3. “Clinical Trial” [Publication Type] NOT(“Clinical Trial, Phase I” [Publication Type] OR “Observational study ” [Publication Type]

#4. “Clinical Trials as Topic” [MeSH] NOT(“Clinical Trial, Phase I” [MeSH] OR “Observational study ” [MeSH]

#5. random*[Title/abstract]

#6. #3 OR #4 OR #5

#7. #1 AND #2 AND #6
