# Supplementary material for: Comparison of Cognitive Intervention Strategies for Individuals With Alzheimer’s Disease: A Systematic Review and Network Meta-analysis
Source: Neuropsychol Rev. 2023 Mar 16;34(2):402–16. doi: 10.1007/s11065-023-09584-5 (PMC11166762; doi:10.1007/s11065-023-09584-5)
Supplement: Supplementary file 2 — Supplementary file2 (DOCX 21 KB) [file 11065_2023_9584_MOESM2_ESM.docx]

| Outcome | Difference between cognitive intervention and controls(95%CI) | No of participants(studies) | Quality of the evidence (GRADE) | Comments |
| --- | --- | --- | --- | --- |
| Global cognition  MMSE | SMD 0.43(0.28 to 0.58) | 2053 (39 RCTs) | ⊕⊕⊕○^a^  Moderate | Cognitive intervention has a modest effect on global cognition |
| ADAS-Cog | SMD -0.33（-0.53 to -0.12) | 374(9 RCTs) | ⊕⊕⊕○^b^  Moderate | Cognitive intervention has a modest effect on global cognition |
| Cognitive domain  Working memory | SMD 0.36（0.11 to 0.61) | 239(7 RCTs) | ⊕⊕⊕○^b^  Moderate | Cognitive intervention has a modest effect on working memory |
| Immediate verbal memory | SMD 0.37（0.12 to 0.62) | 252(5 RCTs) | ⊕⊕⊕○^b^  Moderate | Cognitive intervention has a modest effect on immediate verbal memory |
| Delayed verbal memory | SMD 0.20（0.03 to 0.49) | 294(5 RCTs) | ⊕⊕○○^b,c^  Low | Cognitive intervention may have a modest effect on delayed verbal memory |
| Verbal fluency | SMD 0.26（0.05 to 0.47) | 338(7 RCTs) | ⊕⊕⊕○^b^  Moderate | Cognitive intervention has a modest effect on verbal fluency |
| Confrontation Naming | SMD 0.42（0.18 to 0.66) | 283(7 RCTs) | ⊕⊕⊕○^b^  Moderate | Cognitive intervention has a modest effect on confrontation naming |
| Attention | SMD 0.32（0.03 to 0.62) | 169(6 RCTs) | ⊕⊕⊕○^b^  Moderate | Cognitive intervention has a modest effect on attention |
| Execution | SMD -0.05（0.50 to 0.40) | 70(4 RCTs) | ⊕⊕○○^b, c^  Low | Cognitive intervention may not have an effect on execution |
| Visuospatial skills | SMD 0.61(-0.20 to 1.42) | 92(4 RCTs) | ⊕○○○^a, b, d^  Very Low | We are unable to determine whether there is any effect on visuospatial skills due to very low quality of evidence |
| Processing speed | SMD 0.20(-0.17 to 0.56) | 210(6 RCTs) | ⊕○○○^b, d^  Very Low | We are unable to determine whether there is any effect on visuospatial skills due to very low quality of evidence |
| Immediate non-verbal memory | SMD 0.15(-0.20 to 0.49) | 126(4 RCTs) | ⊕○○○^b, d^  Very Low | We are unable to determine whether there is any effect on immediate non-verbal memory due to very low quality of evidence |
| Delayed non-verbal memory | SMD 0.09(-0.25 to 0.43) | 126(4 RCTs) | ⊕○○○^b, d^  Very Low | We are unable to determine whether there is any effect on delayed non-verbal memory due to very low quality of evidence |
| Non-cognitive domain  Neuropsychiatric symptom | SMD -1.87(-2.89 to -0.85) | 217(4 RCTs) | ⊕○○○^b, e^  Very Low | We are unable to determine whether there is any effect on neuropsychiatric symptom due to very low quality of evidence |
| Depression | SMD -0.24(-0.51 to 0.04) | 629(12 RCTs) | ⊕⊕○○^a, c^  Low | Cognitive intervention may not have an effect on depression |
| Quality of life | SMD 0.35(0.14 to 0.56) | 364(6 RCTs) | ⊕⊕⊕○^b^  Moderate | Cognitive intervention has a modest effect on quality of life |
| Basic activities of daily living | SMD 0.42(0.04 to 0.81) | 288(4 RCTs) | ⊕⊕○○^a, b^  Low | Cognitive intervention may have an effect on basic activities of living |
| Instrumental activities of daily living | SMD 0.21(-0.04 to 0.46) | 219(5 RCTs) | ⊕○○○^b, c^  Low | Cognitive intervention may not have an effect on instrumental activities of living |

a. In terms of inconsistency, degraded 1 point for serious concerns due to moderate heterogeneity（40% to 70%）and when subgroup analyses did not seem to explain the heterogeneity

b. In terms of imprecision, degraded 1 for serious concerns point due to small sample size (fewer than 400 participants)

c. In terms of imprecision, degraded 1 for serious concerns point due to small effect size or the confidence interval does cross the effect threshold (fewer than 0.2 in either direction for continuous outcome)

d. In terms of imprecision, degraded 2 points for serious concerns point due to small effect size and the confidence interval does cross the effect threshold (fewer than 0.2 in either direction for continuous outcome)

a. In terms of inconsistency, degraded 2 points for very serious concerns due to large heterogeneity（more than 70%）and when subgroup analyses did not seem to explain the heterogeneity

**Table S2 Summary of the main comparison**
