# Supplementary material for: Comparison of Cognitive Intervention Strategies for Individuals With Alzheimer’s Disease: A Systematic Review and Network Meta-analysis
Source: Neuropsychol Rev. 2023 Mar 16;34(2):402–16. doi: 10.1007/s11065-023-09584-5 (PMC11166762; doi:10.1007/s11065-023-09584-5)
Supplement: Supplementary file 3 — Supplementary file3 (DOCX 17 KB) [file 11065_2023_9584_MOESM3_ESM.docx]

Table 3. Network league table showing results of network meta-analysis using the mean difference and 95% confidence interval for cognitive interventions based on MMSE

MMSE, Mini-Mental State Examination; CS, cognitive stimulation; CT, cognitive training; CR, cognitive rehabilitation.

| Combined therapy |  |  |  |  |
| --- | --- | --- | --- | --- |
| 0.16 (-0.23,0.54) | CT |  |  |  |
| 0.25 (-0.10,0.60) | 0.09 (-0.28,0.46) | CS |  |  |
| 0.48 (-0.19,1.16) | 0.33 (-0.36,1.01) | 0.24 (-0.43,0.90) | CR |  |
| 0.59 (0.33,0.86) | 0.43 (0.16,0.71) | 0.34 (0.10,0.58) | 0.11 (-0.52,0.73) | Control |

*Comparisons of treatments should be read from left to right. The mean difference lower than 0 favors the top left treatment. The treatments have been sorted from left

to right according to treatment ranking
