# Supplementary material for: Comparison of Cognitive Intervention Strategies for Individuals With Alzheimer’s Disease: A Systematic Review and Network Meta-analysis
Source: Neuropsychol Rev. 2023 Mar 16;34(2):402–16. doi: 10.1007/s11065-023-09584-5 (PMC11166762; doi:10.1007/s11065-023-09584-5)
Supplement: Supplementary file 4 — Supplementary file4 (DOC 98 KB) [file 11065_2023_9584_MOESM4_ESM.doc]

Combined therapy


CT


CR


control
