# Supplementary material for: Comparison of Cognitive Intervention Strategies for Individuals With Alzheimer’s Disease: A Systematic Review and Network Meta-analysis
Source: Neuropsychol Rev. 2023 Mar 16;34(2):402–16. doi: 10.1007/s11065-023-09584-5 (PMC11166762; doi:10.1007/s11065-023-09584-5)
Supplement: Supplementary file 6 — Supplementary file6 (DOC 16 KB) [file 11065_2023_9584_MOESM6_ESM.doc]

Random sequence generation (selection bias) Allocation concealment (selection bias)
Blinding of participants and personnel (pe1formance bias) Blinding of outcome assessment (detection bias) Incomplete outcome data (attrition bias)
Selective reporting (reporting bias)
Other bias
