# Supplementary material for: Comparison of Cognitive Intervention Strategies for Individuals With Alzheimer’s Disease: A Systematic Review and Network Meta-analysis
Source: Neuropsychol Rev. 2023 Mar 16;34(2):402–16. doi: 10.1007/s11065-023-09584-5 (PMC11166762; doi:10.1007/s11065-023-09584-5)
Supplement: Supplementary file 7 — Supplementary file7 (DOC 126 KB) [file 11065_2023_9584_MOESM7_ESM.doc]

."o'
Q)
u
"E'
"'
.o
C:	Q)


."o'
.oC,:


Q)	"'
C:	,-Q,)
."o'

.o
"'	.o,

C:
"'	C:
.,	"'

C:	o
C:	u	Q)
C:
Q)	.o

.o,

Q)	C.
Q)
E
"'	"'	CI

Q)	"'	C:
"Q)'
- t:

Q)	-C:	,",'	C.


C:
CI	Q)
""'	"'	"'	o
Q)	Q)

Q)	E	"C.'
c-

CI
o

"'	oC:
- - -Q)	Q)	"'

,E,	.,	,,
,C,I	C.
."o'

o	"u '
C:	C:
E	u	�
o	Q)	Q)


Amieva 2016

Barban 2015

Bergamaschi 2013

Bottino 2005

Brueggen 2017

Buschert 2011

Cahn-Weiner 2003

Capotosto 2016

Casoli 2020

Cavallo 2016

Cavallo 2018

Cinar 2020

Clare 2019

Coen 2011

Cove 2014

Davis 2001 Fernandez-Calvo2014
Fonte 2019

Giddor 2020

Giovagnoli 2017

Giuli 2016

Huntley 2016

Jelcic 2012

Jelcic 2014

Kallio 2018

Kang 2019

Kim 2015

Kim 2020

Kurz 2011

Lee 2013

Lopez 2020

Luttenberger 2012

Maci 2012

Mapelli 2013 Niu 201O
Nousia 2018

Okamura 2018

Orrell 2014

Spector 2003

Ta'rraga 2006

Thivierge 2014

Trebbastoni 2018

Venturelli 2016

Wang 2007

Yamanaka 2013

Young 2018
o	C:	C:
ct'.	<	CD	CD
u	Q)
C:	00
