# Supplementary material for: Comparison of Cognitive Intervention Strategies for Individuals With Alzheimer’s Disease: A Systematic Review and Network Meta-analysis
Source: Neuropsychol Rev. 2023 Mar 16;34(2):402–16. doi: 10.1007/s11065-023-09584-5 (PMC11166762; doi:10.1007/s11065-023-09584-5)

1. Working memory


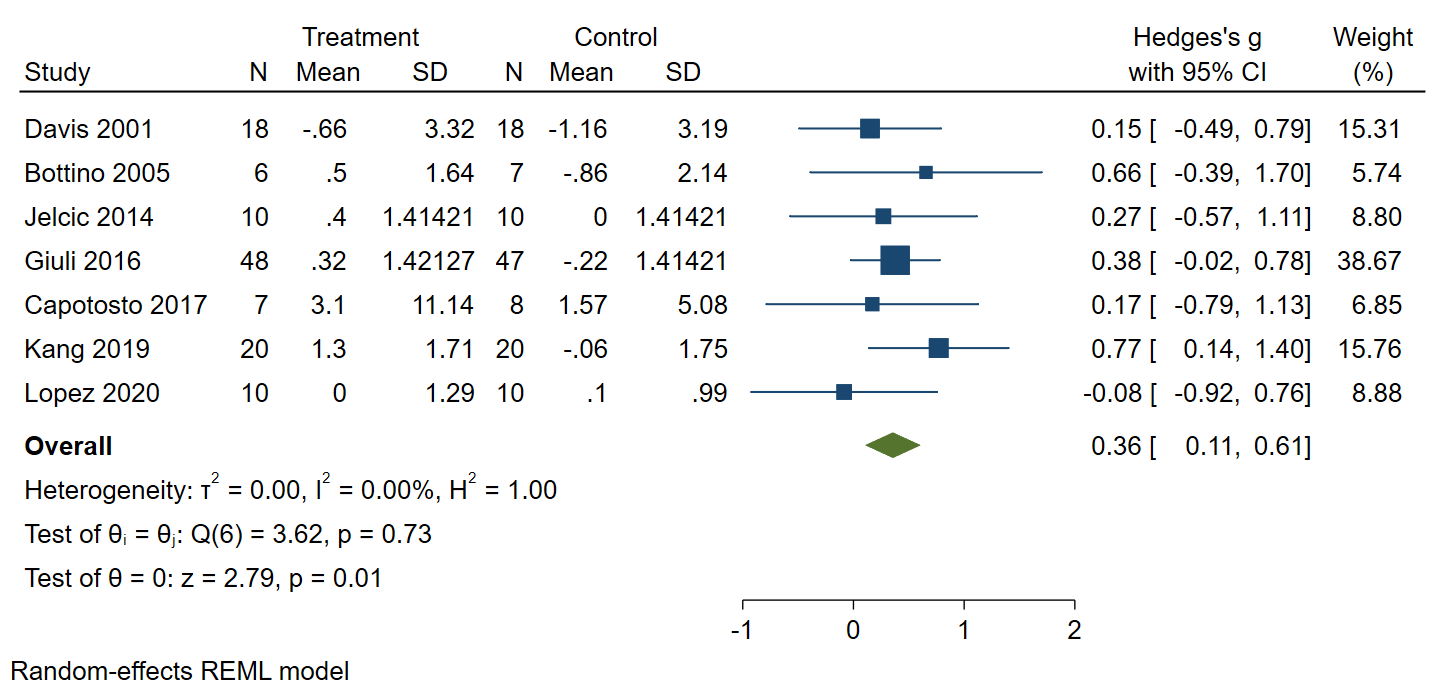


1. Immediate verbal memory


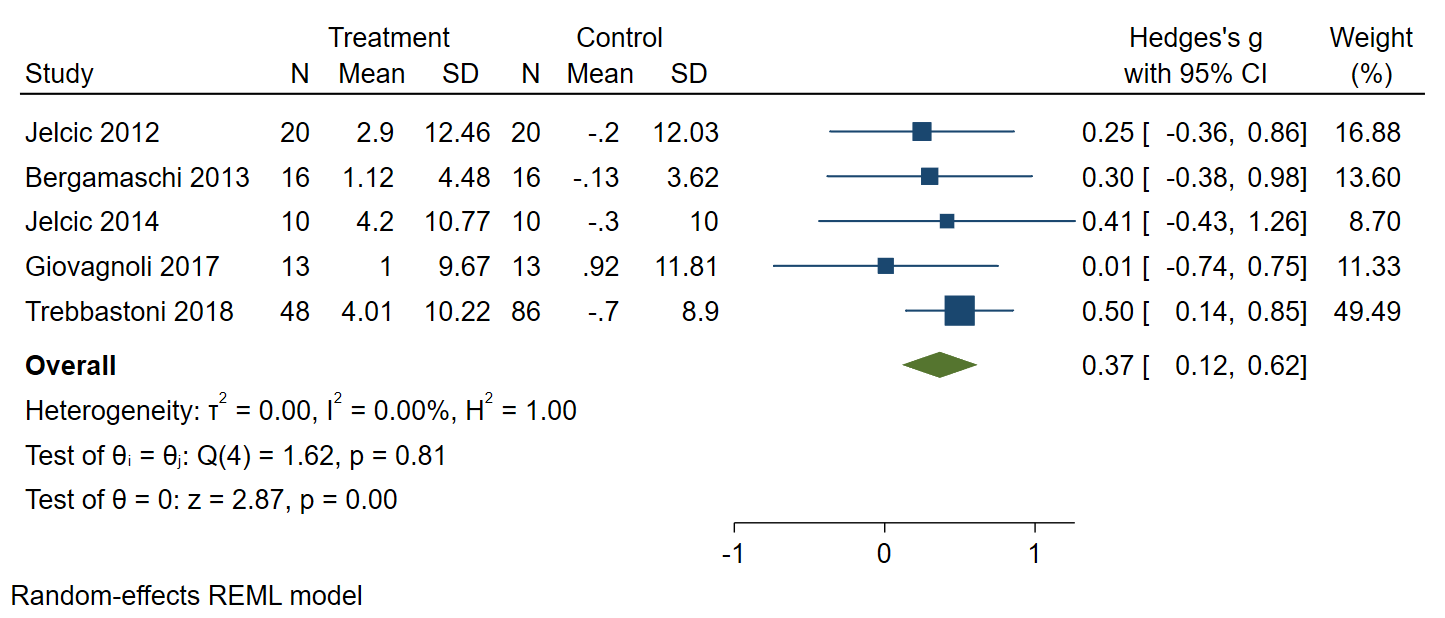


1. Delayed verbal memory


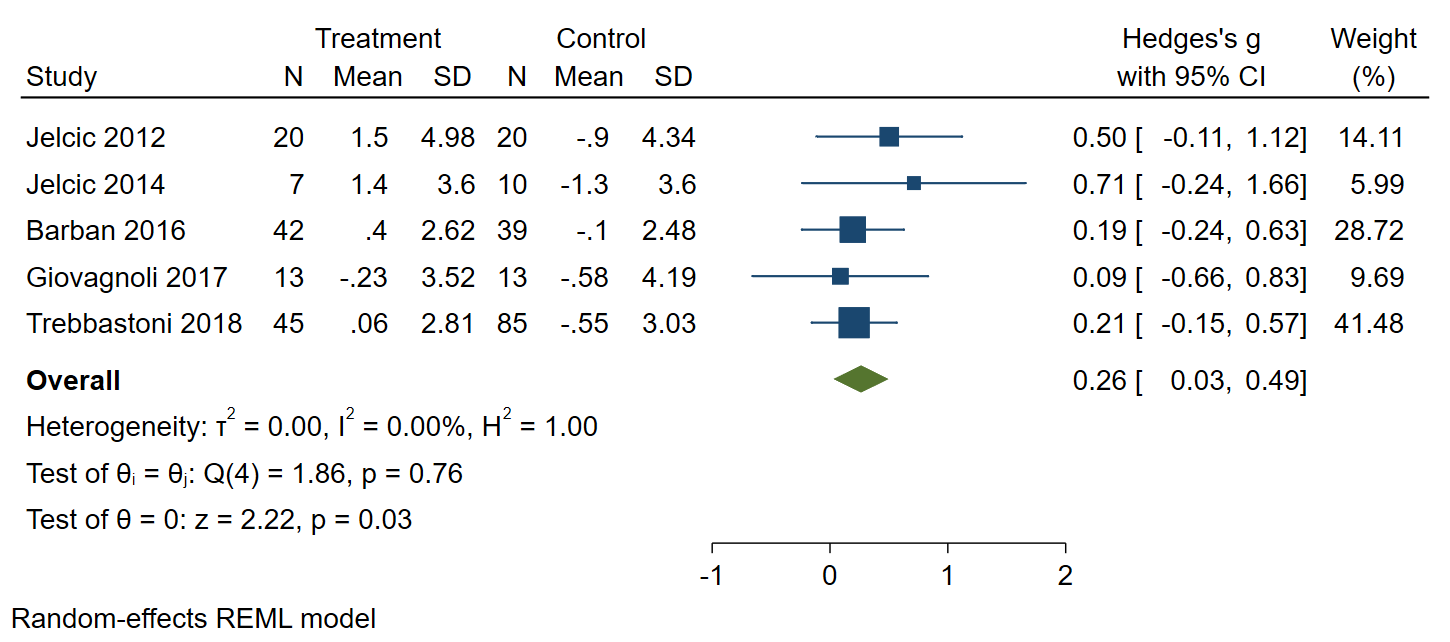


1. Verbal fluency


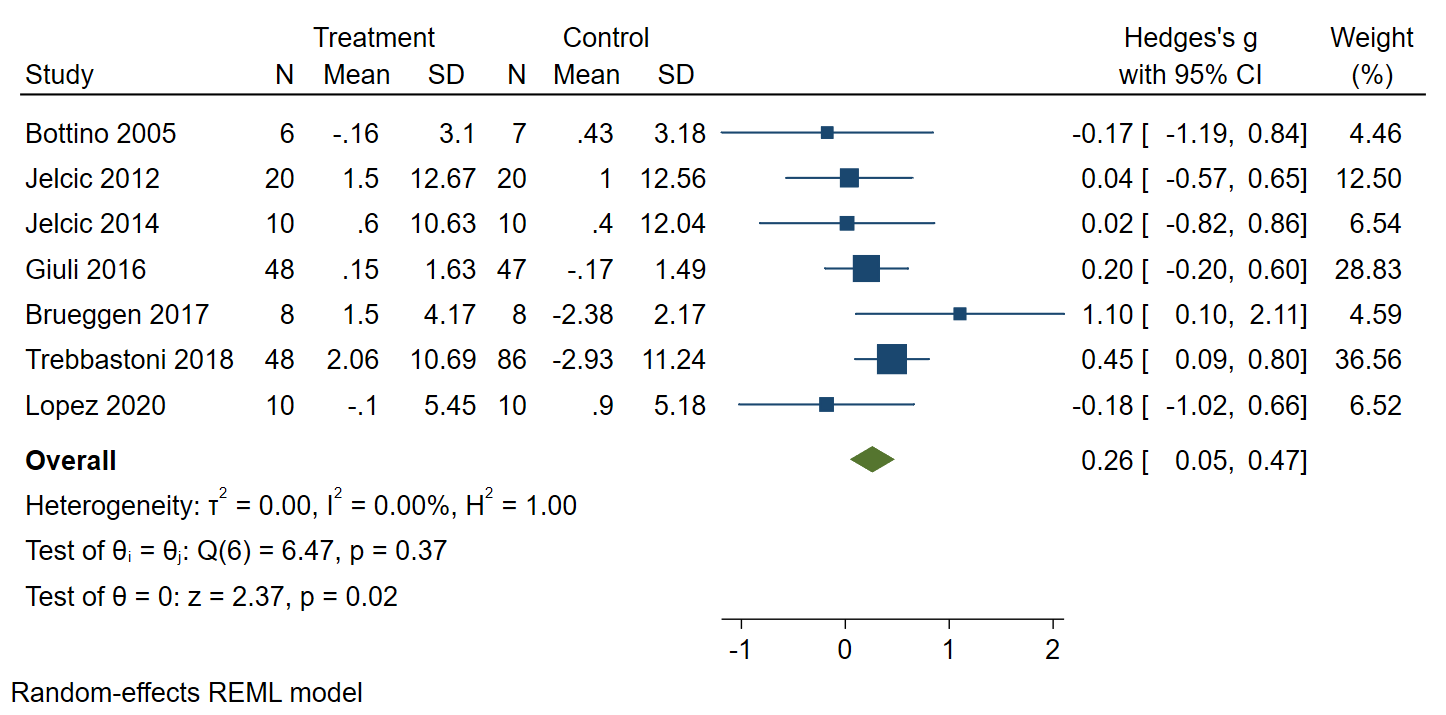


1. Confrontation Naming


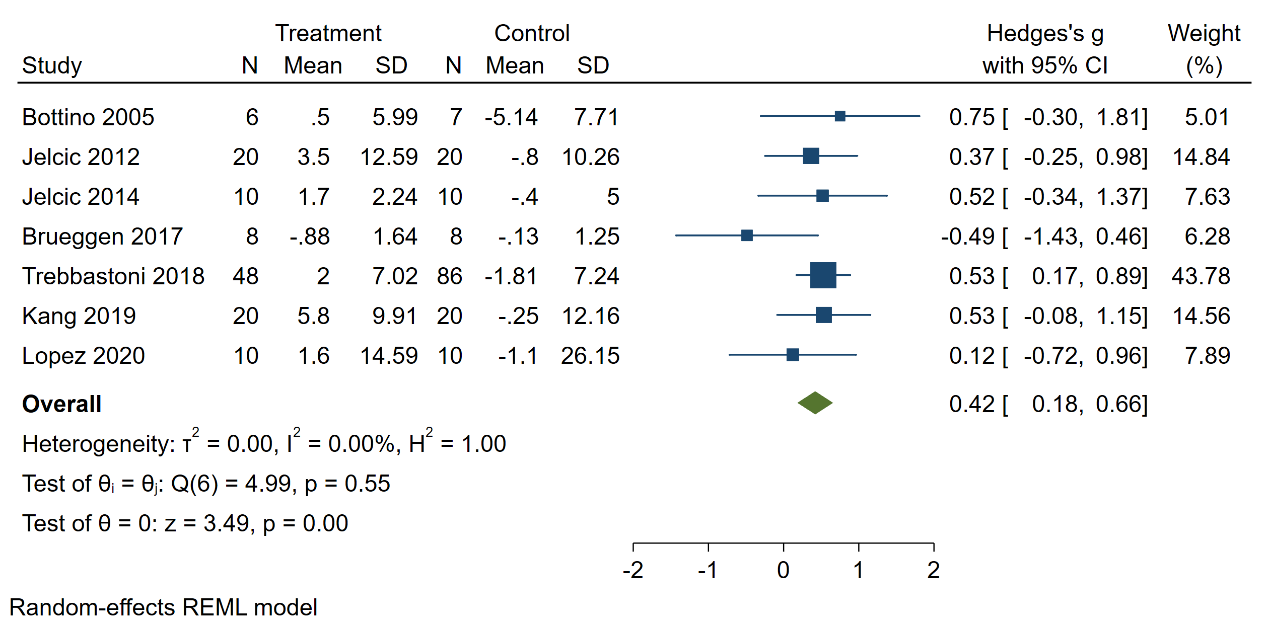


1. Attention


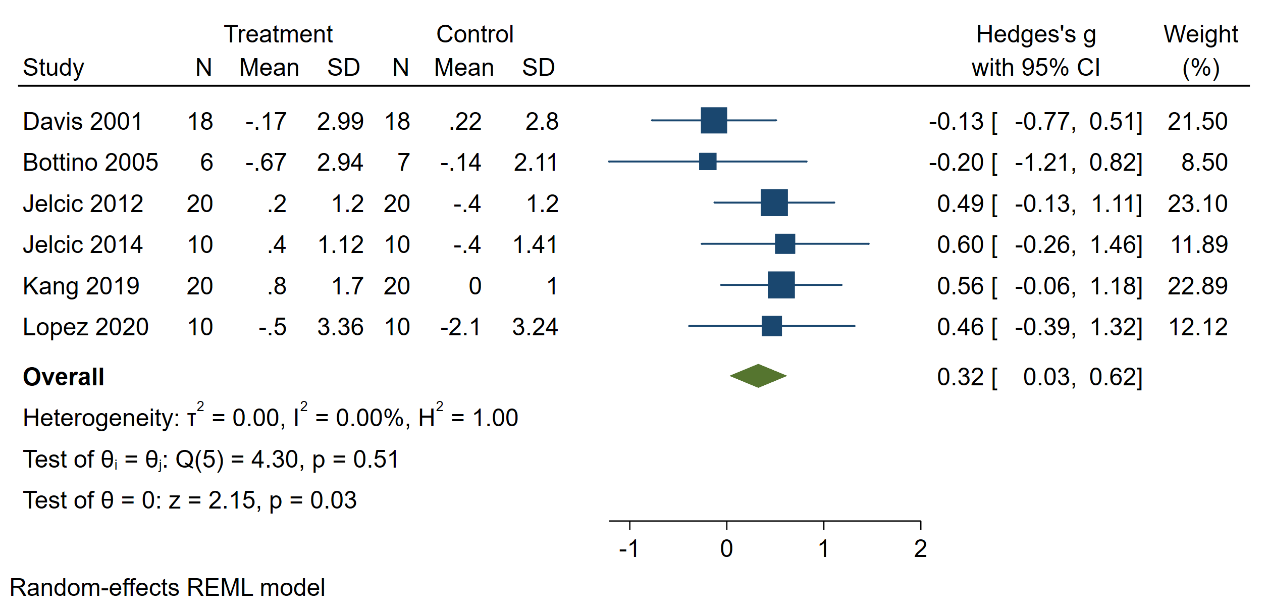

Supplement: Supplementary file 13 — Supplementary file13 (DOCX 600 KB) [file 11065_2023_9584_MOESM13_ESM.docx]
