# Supplementary material for: Comparison of Cognitive Intervention Strategies for Individuals With Alzheimer’s Disease: A Systematic Review and Network Meta-analysis
Source: Neuropsychol Rev. 2023 Mar 16;34(2):402–16. doi: 10.1007/s11065-023-09584-5 (PMC11166762; doi:10.1007/s11065-023-09584-5)
Supplement: Supplementary file 16 — Supplementary file16 (DOCX 244 KB) [file 11065_2023_9584_MOESM16_ESM.docx]

Network meta-analysis

1. Prepare data for network meta-analysis

**network setup mean sd n, study(study) trt(t) ref(control) smd**

Treatments used

A: CR

B: CS

C: CT

D: Combined

E (reference): control

Measure Standardised mean difference

Standard deviation pooling: on

Studies

ID variable: study

Number used: 39

Network information

Components: 1 (connected)

D.f. for inconsistency: 2

D.f. for heterogeneity: 34

Current data

Data format: augmented

Design variable: _design

Estimate variables: _y*

Variance variables: _S*

Command to list the data: list study _y* _S*, noo sepby(_design)

1. Map of network

**network map, improve**

Improving locations ...

loop 1 score 1=.= 0

Stopping after achieving score of 0

Evaluating optimal locations ...

Graph command stored in F9

1. Perform network meta-analysis under inconsistency model

**network meta i**

Command is: mvmeta _y _S , bscovariance(exch 0.5) longparm suppress(uv mm) eq(_y_B: des_BE, _y_D: des_DE) vars(_y_A _y_B _y_C _y

> _D)

Note: using method reml

Note: regressing _y_A on (nothing)

Note: regressing _y_B on des_BE

Note: regressing _y_C on (nothing)

Note: regressing _y_D on des_DE

Note: 39 observations on 4 variables

Note: variance-covariance matrix is proportional to .5*I(4)+.5*J(4,4,1)

initial: log likelihood = -40.745855

rescale: log likelihood = -31.44596

rescale eq: log likelihood = -28.670125

Iteration 0: log likelihood = -28.670125

Iteration 1: log likelihood = -27.291167

Iteration 2: log likelihood = -27.283111

Iteration 3: log likelihood = -27.283105

Iteration 4: log likelihood = -27.283105

Multivariate meta-analysis

Variance-covariance matrix = proportional .5*I(4)+.5*J(4,4,1)

Method = reml Number of dimensions = 4

Restricted log likelihood = -27.283105 Number of observations = 39

------------------------------------------------------------------------------

| Coef. Std. Err. z P>|z| [95% Conf. Interval]

-------------+----------------------------------------------------------------

_y_A |

_cons | .1097379 .3238466 0.34 0.735 -.5249898 .7444655

-------------+----------------------------------------------------------------

_y_B |

des_BE | -.0523633 .5401116 -0.10 0.923 -1.110963 1.006236

_cons | .397295 .5238864 0.76 0.448 -.6295036 1.424094

-------------+----------------------------------------------------------------

_y_C |

_cons | .4343617 .144969 3.00 0.003 .1502277 .7184957

-------------+----------------------------------------------------------------

_y_D |

des_DE | -.145542 .5518534 -0.26 0.792 -1.227155 .9360707

_cons | .7239056 .5323626 1.36 0.174 -.3195059 1.767317

------------------------------------------------------------------------------

Estimated between-studies SDs and correlation matrix:

SD _y_A _y_B _y_C _y_D

_y_A .35575378 1 . . .

_y_B .35575378 .5 1 . .

_y_C .35575378 .5 .5 1 .

_y_D .35575378 .5 .5 .5 1

Testing for inconsistency:

( 1) [_y_B]des_BE = 0

( 2) [_y_D]des_DE = 0

chi2( 2) = 0.07

Prob > chi2 = 0.9650

mvmeta command stored as F9; test command stored as F8

1. Perform network meta-analysis under consistency model

**network meta c**

Command is: mvmeta _y _S , bscovariance(exch 0.5) longparm suppress(uv mm) vars(_y_A _y_B _y_C _y_D)

Note: using method reml

Note: using variables _y_A _y_B _y_C _y_D

Note: 39 observations on 4 variables

Note: variance-covariance matrix is proportional to .5*I(4)+.5*J(4,4,1)

initial: log likelihood = -42.692265

rescale: log likelihood = -32.323801

rescale eq: log likelihood = -28.888681

Iteration 0: log likelihood = -28.888681

Iteration 1: log likelihood = -27.791117

Iteration 2: log likelihood = -27.78076

Iteration 3: log likelihood = -27.78075

Iteration 4: log likelihood = -27.78075

Multivariate meta-analysis

Variance-covariance matrix = proportional .5*I(4)+.5*J(4,4,1)

Method = reml Number of dimensions = 4

Restricted log likelihood = -27.78075 Number of observations = 39

------------------------------------------------------------------------------ | Coef. Std. Err. z P>|z| [95% Conf. Interval]

-------------+----------------------------------------------------------------

_y_A |

_cons | .1062173 .3183748 0.33 0.739 -.5177858 .7302205

-------------+----------------------------------------------------------------

_y_B |

_cons | .3423876 .1232664 2.78 0.005 .1007898 .5839854

-------------+----------------------------------------------------------------

_y_C |

_cons | .43354 .1417856 3.06 0.002 .1556453 .7114347

-------------+----------------------------------------------------------------

_y_D |

_cons | .5905099 .1351674 4.37 0.000 .3255866 .8554332

------------------------------------------------------------------------------

Estimated between-studies SDs and correlation matrix:

SD _y_A _y_B _y_C _y_D

y_A .34212008 1 . . .

y_B .34212008 .5 1 . .

y_C .34212008 .5 .5 1 .

y_D .34212008 .5 .5 .5 1

1. Generalizes the forest plot for pairwise meta-analysis.

**network forest**


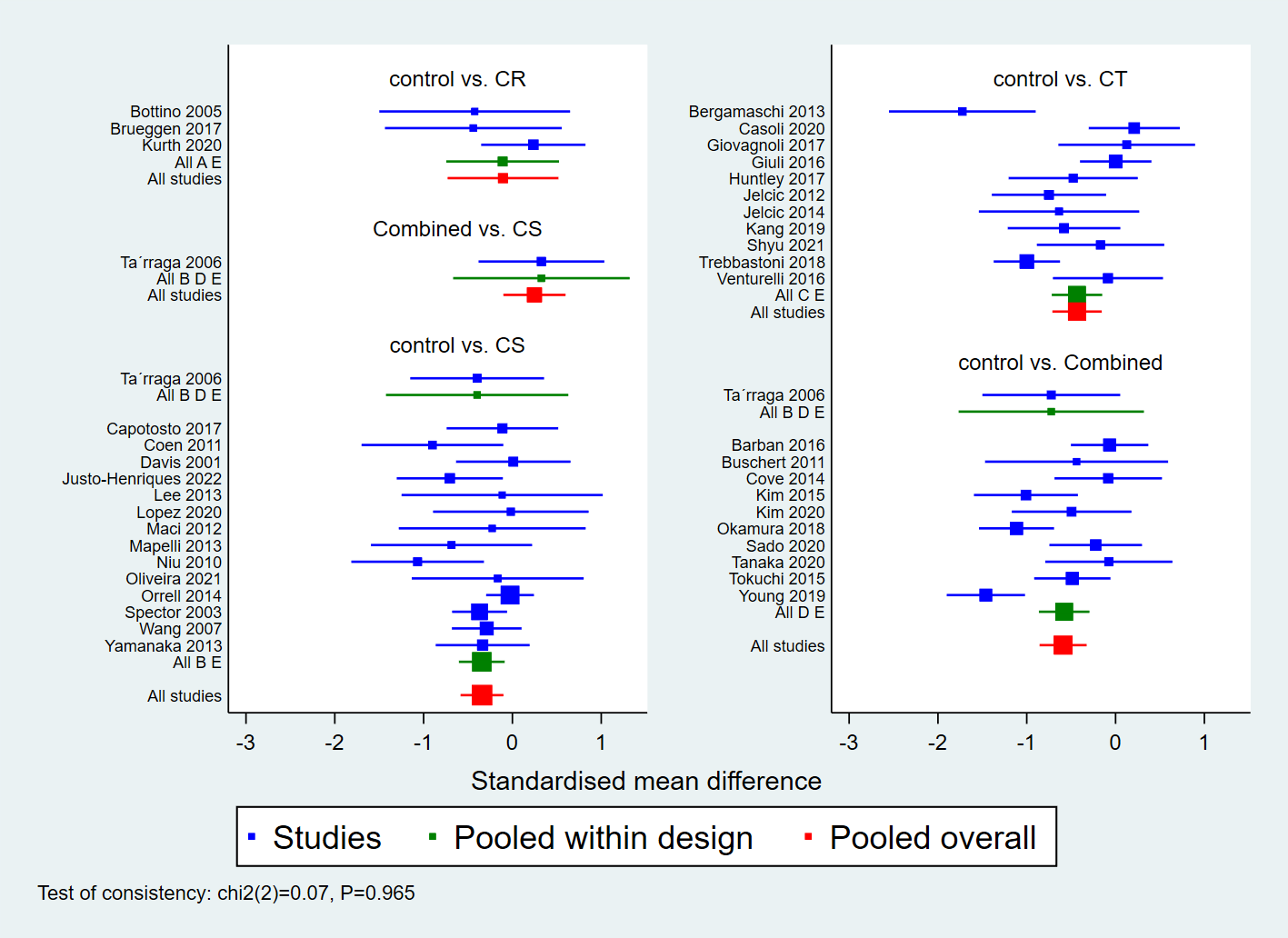


1. Fit side-splitting model

**network sidesplit all, tau**

Side Direct Indirect Difference tau

Coef. Std. Err. Coef. Std. Err. Coef. Std. Err. P>|z|

AE . . . . . . . .B E * -.3474918 .1260531 -.112487 .9340929 -.2350047 .9443005 0.803 .3490122

B D .3246048 .5025522 .2354549 .1940129 .0891499 .5386402 0.869 .3494116

CE . . . . . . . .D E * -.5890163 .1386236 -.6264977 .9123864 .0374814 .9242236 0.968 .3492086

* Warning: all the evidence about these contrasts comes from the trials which directly compare them.

See help file for more information.

1. SUCRA: rank treatments after network meta-analysis

**network rank max, all zero reps(5000) gen(prob)**

**sucra prob*,lab(control CR CS CT combined)**

Command is: mvmeta, noest pbest(max in 1, zero id(study) all reps(5000) gen(prob) stripprefix(_y_) zeroname(E) rename(A = CR, B

> = CS, C = CT, D = Combined, E = control))

Estimated probabilities (%) of each treatment being the best (and other ranks)

- assuming the maximum parameter is the best

- using 5000 draws

- allowing for parameter uncertainty

--------------------------------------------------------------

study and | Treatment

Rank | control CR CS CT Combined

------------+-------------------------------------------------

Barban 2016 |

Best | 0.0 6.9 4.7 17.6 70.8

2nd | 0.0 9.3 24.2 44.4 22.0

3rd | 0.1 12.6 51.4 29.5 6.3

4th | 36.6 34.6 19.5 8.4 0.8

Worst | 63.2 36.5 0.2 0.1 0.0

--------------------------------------------------------------

mvmeta command is stored in F9

.

. sucra prob*,lab(control CR CS CT combined)

Treatment Relative Ranking of Model 1

+--------------------------------------+

| Treatm~t | SUCRA | PrBest | MeanRank |

|----------+-------+--------+----------|

| control | 9.2 | 0.0 | 4.6 |

| CR | 28.9 | 6.9 | 3.8 |

| CS | 53.4 | 4.7 | 2.9 |

| CT | 67.8 | 17.6 | 2.3 |

| combined | 90.7 | 70.8 | 1.4 |

|  |
| --- |

1. Head-to-head comparison

**netleague, lab(control CR CS CT combined) sort(combined CT CS CR control)**

**intervalplot, null(0) lab(control CR CS CT combined)**

--------------------------------------------------------------------------------------------------+

| _Comparison | _Effect_Size | _Standard_Error | _LCI | _UCI | _LPrI | _UPrI |

|---------------------+--------------+-----------------+-----------+----------+-----------+----------|

| CR vs control | .1062173 | .3183748 | -.5177858 | .7302205 | -.5177858 | .7302205 |

| CS vs control | .3423876 | .1232664 | .1007898 | .5839854 | .1007898 | .5839854 |

| CT vs control | .43354 | .1417856 | .1556453 | .7114347 | .1556453 | .7114347 |

| combined vs control | .5905099 | .1351674 | .3255866 | .8554332 | .3255866 | .8554332 |

| CS vs CR | .2361703 | .3411152 | -.4324033 | .9047439 | -.4324033 | .9047439 |

| CT vs CR | .3273227 | .3482955 | -.3553239 | 1.009969 | -.3553239 | 1.009969 |

| combined vs CR | .4842925 | .3461997 | -.1942464 | 1.162832 | -.1942464 | 1.162832 |

| CT vs CS | .0911524 | .1877559 | -.2768423 | .4591472 | -.2768423 | .4591472 |

| combined vs CS | .2481223 | .1787875 | -.1022948 | .5985394 | -.1022948 | .5985394 |

| combined vs CT | .1569699 | .1960215 | -.2272252 | .5411649 | -.2272252 | .5411649 |

|  |
| --- |

1. Netfunnel

Comparison-adjusted funnel plot for a network of interventions

**network convert pairs**

**netfunnel _y _stderr _t1 _t2 , random bycomp add(lfit _stderr _ES_CEN) noalpha**

**
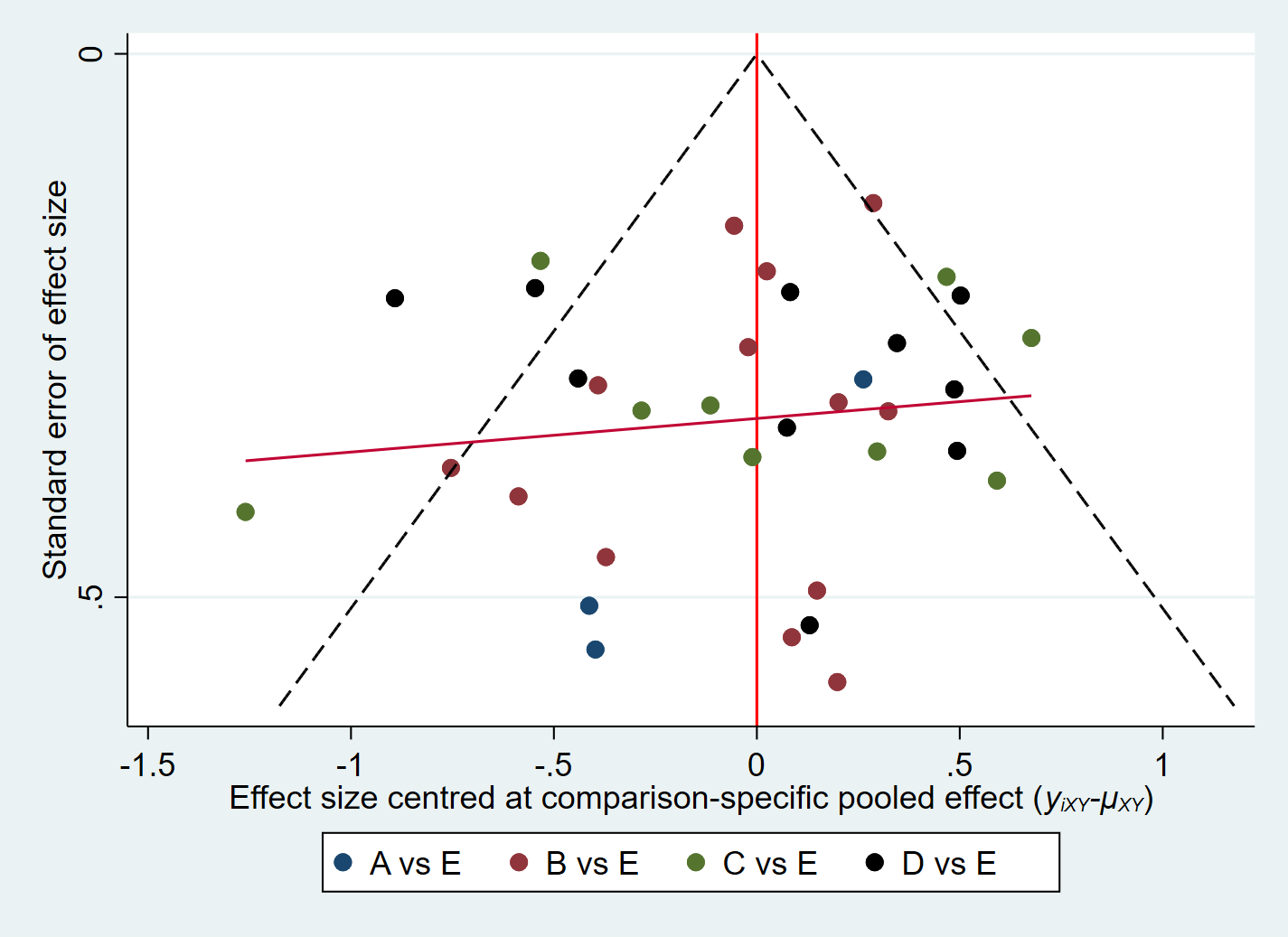
**

1. loop inconsistency

**ifplot _y _stderr _t1 _t2 study, tau2(loop)**

* 1 triangular loops found

Evaluation of inconsistency using loop-specific heterogeneity estimates:

+-----------------------------------------------------------------------------+

| Loop | IF | seIF | z_value | p_value | CI_95 | Loop_Heterog_tau2 |

|-------+-------+-------+---------+---------+-------------+-------------------|

| B-D-E | 0.123 | 0.444 | 0.278 | 0.781 | (0.00,0.99) | 0.042 |

+-----------------------------------------------------------------------------+
