# Supplementary material for: Comparison of Cognitive Intervention Strategies for Individuals With Alzheimer’s Disease: A Systematic Review and Network Meta-analysis
Source: Neuropsychol Rev. 2023 Mar 16;34(2):402–16. doi: 10.1007/s11065-023-09584-5 (PMC11166762; doi:10.1007/s11065-023-09584-5)
Supplement: Supplementary file 18 — Supplementary file18 (DOCX 18 KB) [file 11065_2023_9584_MOESM18_ESM.docx]

1. Pairwise meta-analysis

**meta esize ne meane sde nc meanc sdc, studylabel(study)**

Meta-analysis setting information

Study information

No. of studies: 39

Study label: study

Study size: _meta_studysize

Summary data: ne meane sde nc meanc sdc

Effect size

Type: hedgesg

Label: Hedges's g

Variable: _meta_es

Bias correction: Approximate

Precision

Std. Err.: _meta_se

Std. Err. adj.: None

CI: [_meta_cil, _meta_ciu]

CI level: 95%

Model and method

Model: Random-effects

Method: REML

1. Forestplot

**meta forestplot, random(reml)**

Effect-size label: Hedges's g

Effect size: _meta_es

Std. Err.: _meta_se

Study label: study

1. Sensivity analysis

**ssc install metaninf**

**metaninf ne meane sde nc meanc sdc, label(namevar=study)**

------------------------------------------------------------------------------

Study omitted | Estimate [95% Conf. Interval]

-------------------+----------------------------------------------------------

Barban 2016 | .43697944 .34587917 .52807969

Bergamaschi 2013 | .40558177 .31588215 .4952814

Bottino 2005 | .4212988 .33181554 .51078206

Brueggen 2017 | .42116627 .33163175 .51070082

Buschert 2011 | .42118174 .33167148 .510692

Capotosto 2017 | .42782155 .33773577 .51790732

Casoli 2020 | .44134888 .35079327 .53190452

Coen 2011 | .41509518 .32535839 .50483197

Cove 2014 | .42901266 .33885413 .51917118

Davis 2001 | .42994478 .33990499 .5199846

Giovagnoli 2017 | .42902666 .33924776 .51880556

Giuli 2016 | .44340056 .3519502 .5348509

Huntley 2017 | .42047516 .33062258 .51032776

Jelcic 2012 | .41478735 .3247436 .50483114

Jelcic 2014 | .41912764 .32951537 .50873989

Justo-Henriques 2022| .41478509 .32460117 .504969

Kang 2019 | .41808069 .32801083 .50815052

Kim 2015 | .40718654 .31695876 .49741435

Kim 2020 | .42000869 .33004361 .50997376

Kurth 2020 | .43712664 .34690556 .52734774

Lee 2013 | .42336896 .3339172 .51282072

Lopez 2020 | .42573571 .33609638 .51537502

Maci 2012 | .42281213 .33331585 .51230836

Mapelli 2013 | .41865018 .32904196 .5082584

Niu 2010 | .41176516 .32194743 .50158286

Okamura 2018 | .38875568 .29752517 .47998616

Oliveira 2021 | .42363653 .33408138 .51319164

Orrell 2014 | .47015098 .37564221 .56465971

Sado 2020 | .42738292 .33687645 .51788938

Shyu 2021 | .4254083 .33553651 .51528007

Spector 2003 | .42604125 .33293095 .51915157

Tanaka 2020 | .42693952 .33706543 .51681358

Ta´rraga 2006 | .41608483 .32634372 .50582594

Tokuchi 2015 | .41838115 .3272216 .50954068

Trebbastoni 2018 | .38624638 .29441404 .47807872

Venturelli 2016 | .42856991 .33845907 .51868075

Wang 2007 | .4286114 .33703843 .5201844

Yamanaka 2013 | .42391202 .3334434 .51438063

Young 2019 | .37667224 .28561401 .46773046

-------------------+----------------------------------------------------------

Combined | .42152154 .3323473 .51069579

------------------------------------------------------------------------------

.

1. Publication analysis

**meta bias, egger random(reml)**

Effect-size label: Hedges's g

Effect size: _meta_es

Std. Err.: _meta_se

Regression-based Egger test for small-study effects

Random-effects model

Method: REML

H0: beta1 = 0; no small-study effects

beta1 = 0.13

SE of beta1 = 0.784

z = 0.16

Prob > |z| = 0.8717
