# Supplementary material for: Comparison of Cognitive Intervention Strategies for Individuals With Alzheimer’s Disease: A Systematic Review and Network Meta-analysis
Source: Neuropsychol Rev. 2023 Mar 16;34(2):402–16. doi: 10.1007/s11065-023-09584-5 (PMC11166762; doi:10.1007/s11065-023-09584-5)
Supplement: Supplementary file 11 [file 11065_2023_9584_MOESM11_ESM.doc]

	Treatment			Control			Hedges's g	Weight
Study	N	Mean	SD	N	Mean	SD	with 95% CI	(%)
Niu 2010	16	-2.06	1.39	16	0	1.03	-1.64 [-2.43, -0.86]	23.96
Fernández-Calvo 2015	25	-1.16	4.64	30	3.16	3.42	-1.06 [-1.62, -0.50]	25.77
Fonte 2019	19	-4	11.39	21	13.6	13.1	-1.40 [-2.08, -0.72]	24.84
Kurth 2020	50	-.9	1.44	50	3.66	1.25	-3.36 [-3.96, -2.75]	25.43
**Overall**							-1.87 [-2.89, -0.85]	

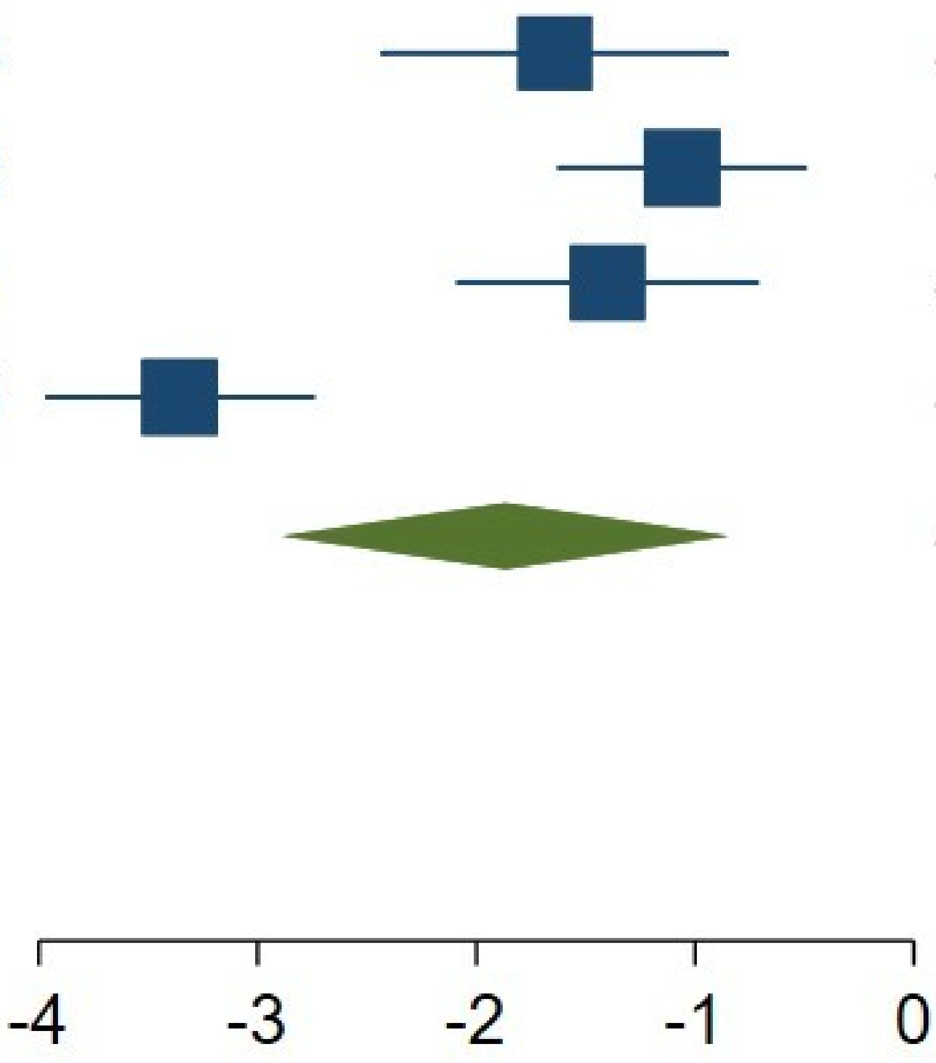

Heterogeneity: $\tau^2 = 0.97$, $I^2 = 89.83\%$, $H^2 = 9.84$

Test of $\theta_i = \theta_j$: Q(3) = 33.28, p = 0.00

Test of $\theta = 0$: z = -3.59, p = 0.00

Random-effects REML model
