# Supplementary material for: Comparison of Cognitive Intervention Strategies for Individuals With Alzheimer’s Disease: A Systematic Review and Network Meta-analysis
Source: Neuropsychol Rev. 2023 Mar 16;34(2):402–16. doi: 10.1007/s11065-023-09584-5 (PMC11166762; doi:10.1007/s11065-023-09584-5)
Supplement: Supplementary file 12 [file 11065_2023_9584_MOESM12_ESM.doc]

Study	Treatment N	Treatment Mean	Treatment SD	Control N	Control Mean	Control SD	Hedges's g with 95% CI	Weight (%)
Spector 2003	97	1.3	5.1	70	-.8	5.6	0.39 [0.08, 0.70]	44.78
Coen 2011	14	3.6	3.7	13	.5	4.4	0.74 [-0.02, 1.50]	7.43
Yamanaka 2013	26	.19	3.48	30	-.43	9.18	0.09 [-0.43, 0.60]	15.92
Kim 2016	32	.4	.76	21	.23	.73	0.22 [-0.32, 0.77]	14.44
Capotosto 2017	7	1.25	11.5	8	.05	9.91	0.11 [-0.85, 1.06]	4.68
Justo-Henriques 2022	22	1.64	7.14	24	-2.5	8.28	0.52 [-0.05, 1.10]	12.76
**Overall**							0.35 [0.14, 0.56]	

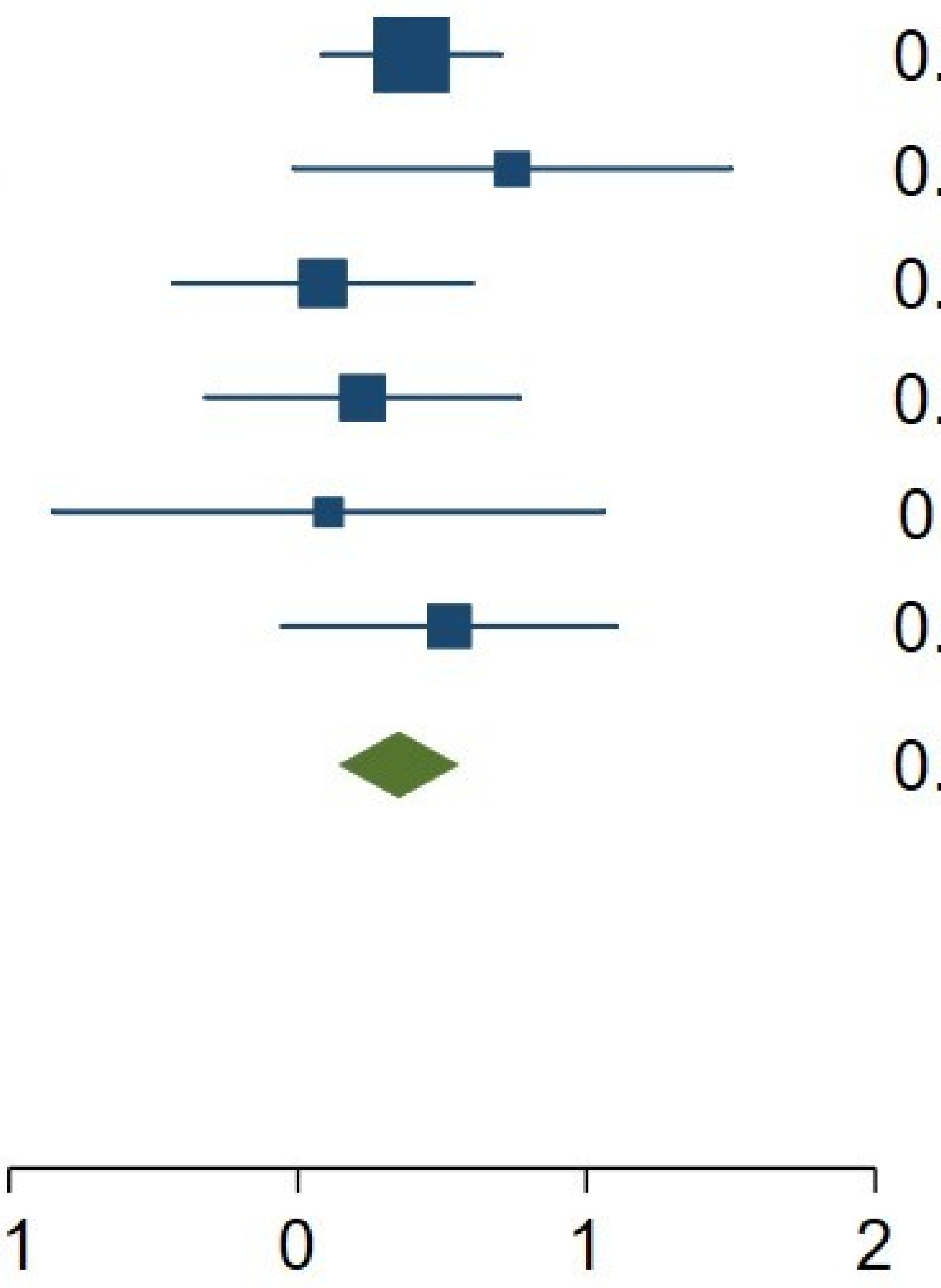

Heterogeneity: $\tau^2 = 0.00$, $I^2 = 0.00\%$, $H^2 = 1.00$

Test of $\theta_i = \theta_j$: Q(5) = 2.91, p = 0.71

Test of $\theta = 0$: z = 3.31, p = 0.00

Random-effects REML model
