# Supplementary material for: Comparison of Cognitive Intervention Strategies for Individuals With Alzheimer’s Disease: A Systematic Review and Network Meta-analysis
Source: Neuropsychol Rev. 2023 Mar 16;34(2):402–16. doi: 10.1007/s11065-023-09584-5 (PMC11166762; doi:10.1007/s11065-023-09584-5)
Supplement: Supplementary file 13 [file 11065_2023_9584_MOESM15_ESM.doc]

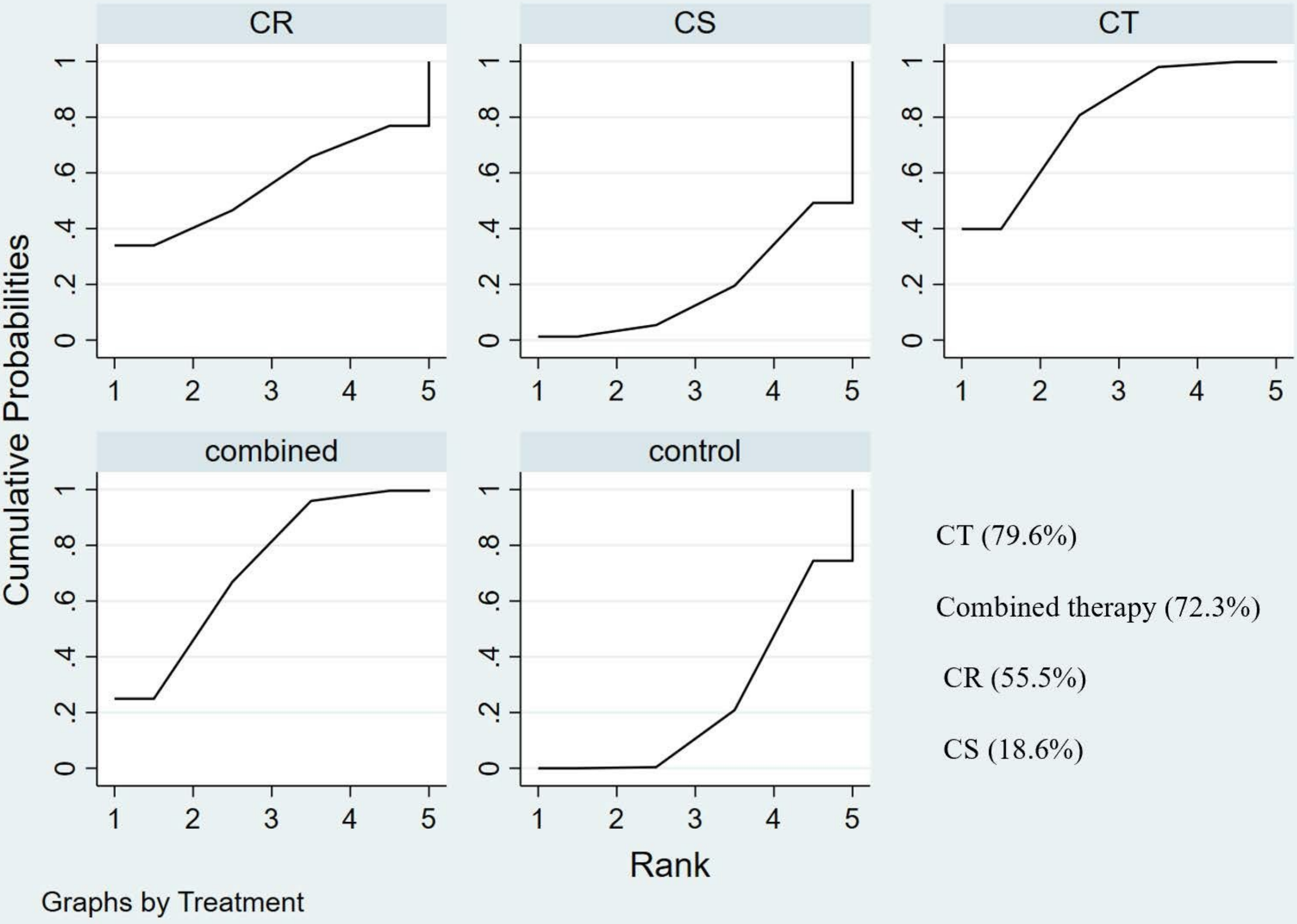
CR
CS
CT
combined
control
Cumulative Probabilities
Rank
0
.2
.4
.6
.8
1
1
2
3
4
5
CT (79.6%)
Combined therapy (72.3%)
CR (55.5%)
CS (18.6%)
Graphs by Treatment
