# Supplementary material for: Comparison of Cognitive Intervention Strategies for Individuals With Alzheimer’s Disease: A Systematic Review and Network Meta-analysis
Source: Neuropsychol Rev. 2023 Mar 16;34(2):402–16. doi: 10.1007/s11065-023-09584-5 (PMC11166762; doi:10.1007/s11065-023-09584-5)
Supplement: Supplementary file 14 [file 11065_2023_9584_MOESM8_ESM.doc]

Study	Treatment N	Treatment Mean	Treatment SD	Control N	Control Mean	Control SD	Hedges's g with 95% CI	Weight (%)
bottino 2005	6	-2.17	8.33	7	.43	8.92	-0.28 [-1.30, 0.74]	3.90
Ta´rraga 2006	16	-2.44	7.22	12	1.08	6.27	-0.50 [-1.24, 0.24]	7.43
Coen 2011	14	-.2	7.2	13	-2.3	4.1	0.34 [-0.39, 1.08]	7.45
Luttenberger 2012	31	-.1	19.14	30	5.2	22.54	-0.25 [-0.75, 0.25]	16.37
Giuli 2016	48	-3.3	13.08	47	-.01	13.3	-0.25 [-0.65, 0.15]	25.27
Capotosto 2017	7	-.9	16.03	8	2.68	17.73	-0.20 [-1.16, 0.76]	4.42
Fonte 2019	30	-1.6	10.68	30	8.1	13.29	-0.79 [-1.31, -0.27]	15.03
Lopez 2020	10	1.8	11.53	10	2.3	13.53	-0.04 [-0.88, 0.80]	5.75
Fernández-Calvo 2014	25	-.52	5	30	2.03	5.45	-0.48 [-1.01, 0.05]	14.38
**Overall**							-0.33 [-0.53, -0.12]	

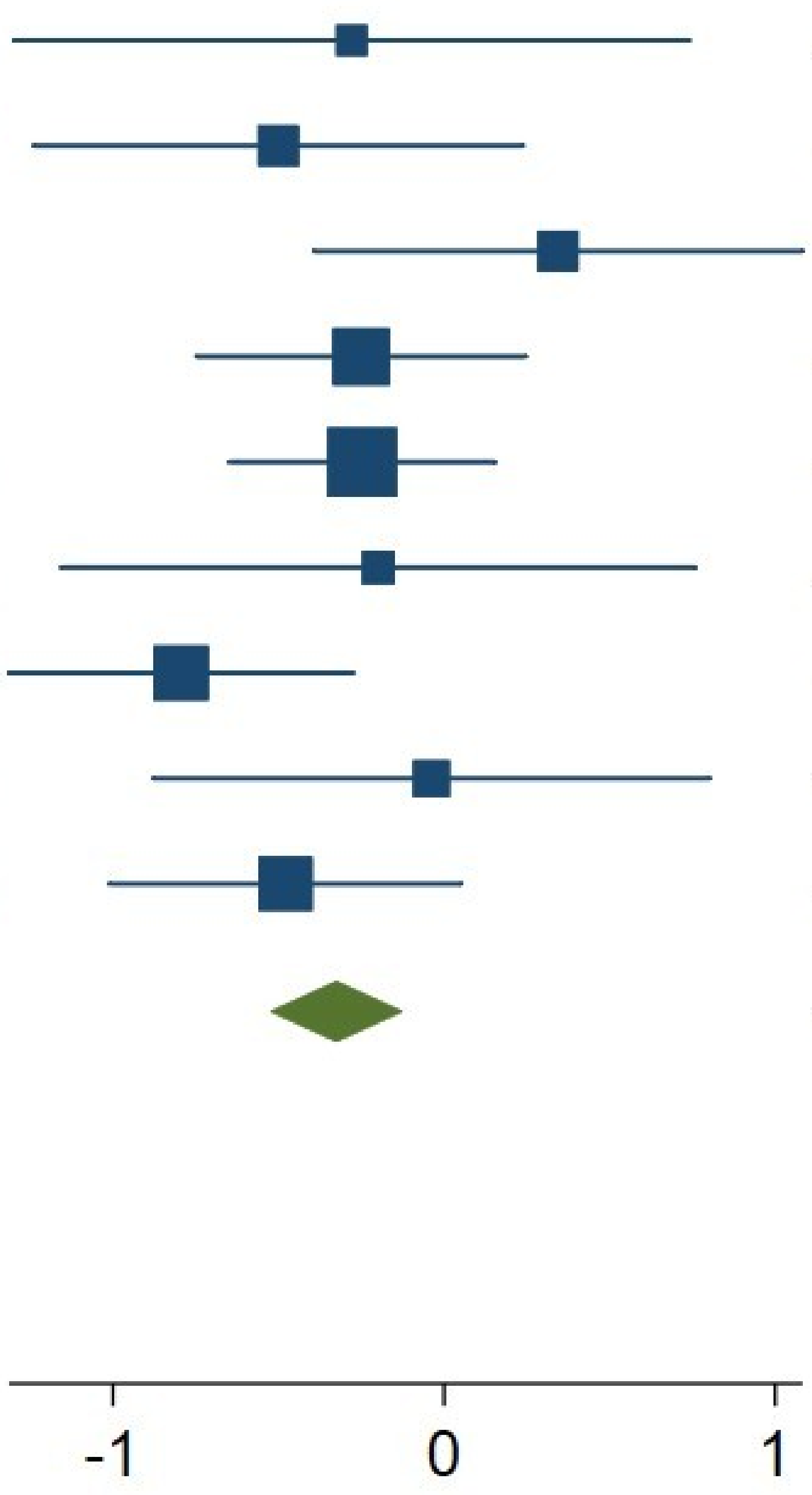

Heterogeneity: $\tau^2 = 0.00$, $I^2 = 0.01\%$, $H^2 = 1.00$

Test of $\theta_i = \theta_j$: $Q(8) = 7.59$, $p = 0.47$

Test of $\theta = 0$: $z = -3.17$, $p = 0.00$

Random-effects REML model
