# Supplementary material for: Comparison of Cognitive Intervention Strategies for Individuals With Alzheimer’s Disease: A Systematic Review and Network Meta-analysis
Source: Neuropsychol Rev. 2023 Mar 16;34(2):402–16. doi: 10.1007/s11065-023-09584-5 (PMC11166762; doi:10.1007/s11065-023-09584-5)
Supplement: Supplementary file 15 [file 11065_2023_9584_MOESM9_ESM.doc]

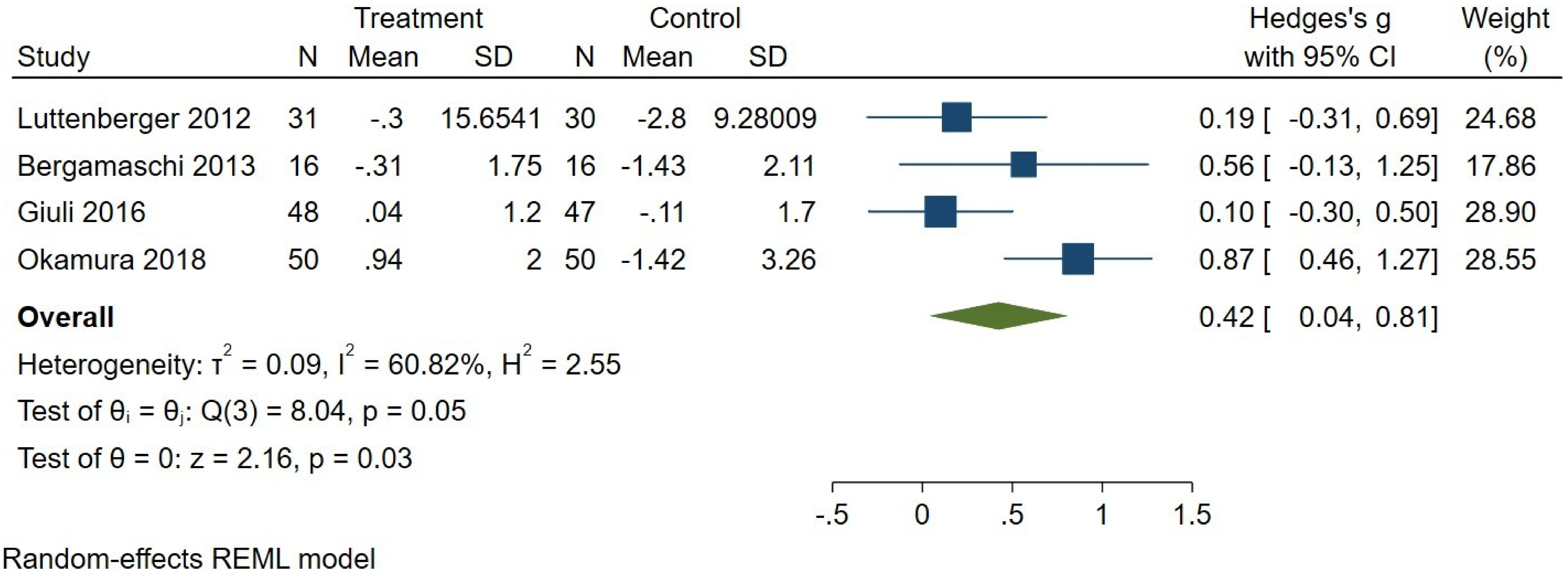
Treatment
Control
Hedges's g with 95% CI
Weight (%)
Study
N
Mean
SD
N
Mean
SD
Luttenberger 2012 31 -.3 15.6541 30 -2.8 9.28009 0.19 [-0.31, 0.69] 24.68
Bergamaschi 2013 16 -.31 1.75 16 -1.43 2.11 0.56 [-0.13, 1.25] 17.86
Giuli 2016 48 .04 1.2 47 -.11 1.7 0.10 [-0.30, 0.50] 28.90
Okamura 2018 50 .94 2 50 -1.42 3.26 0.87 [0.46, 1.27] 28.55
Overall 0.42 [0.04, 0.81]
Heterogeneity: τ^2 = 0.09, I^2 = 60.82%, H^2 = 2.55
Test of $\theta_i = \theta_j$: Q(3) = 8.04, p = 0.05
Test of θ = 0: z = 2.16, p = 0.03
-.5
0
.5
1
1.5
Random-effects REML model
